# A N7-methylguanosine modified circular RNA, circIPP2A2, promotes malignant behaviors in hepatocellular carcinoma by serving as a scaffold in modulating the Hornerin/PI3K/AKT/GSK3β axis

**DOI:** 10.1038/s41419-024-07248-7

**Published:** 2024-11-30

**Authors:** Zeyi Guo, Zhongzhe Li, Jinhao Guo, Luxiang Gan, Haiyu Mo, Jiajun Zhang, Yu Fu, Yi Wang, Meixian Jin, Yanping Wu, Qingyu Xie, Kunjiang Tan, Chunming Wang, Yuyan Xu, Guolin He, Lei Cai, Yi Gao, Mingxin Pan, Shunjun Fu

**Affiliations:** 1 Department of Hepatobiliary Surgery II, Zhujiang Hospital, Southern Medical University, 510000 Guangzhou, Guangdong P. R. China; 2 The Eastern Hepatobiliary Surgery Hospital, Naval Medical University, 200000 Shanghai, P. R. China; 3 Research Centre for Artificial Organ and Tissue Engineering & Institute of Regenerative Medicine, Zhujiang Hospital, Southern Medical University, 510000 Guangzhou, P. R. China; 4 Department of Medical Oncology, Sun Yat-sen University Cancer Center, State Key Laboratory of Oncology in South China, Collaborative Innovation Center for Cancer Medicine, 510000 Guangzhou, P. R. China; 5 Shanxi Cancer Hospital/Shanxi Hospital Affiliated to Cancer Hospital, Chinese Academy of Medical Sciences/Cancer Hospital Affiliated to Shanxi Medical University, 030013 Taiyuan, P. R. China; 6 Department of Anesthesiology of the Second Affiliated Hospital, School of Medicine, The Chinese University of Hong Kong, Shenzhen & Longgang District People’s Hospital of Shenzhen, 518172 Shenzhen, P. R. China; 7 Center of Pancreas, Guangdong Provincial People’s Hospital, Southern Medical University, 510000 Guangzhou, P. R. China

**Keywords:** Non-coding RNAs, Cancer epigenetics

## Abstract

Despite the advancements in treatment strategies, the long-term survival of hepatocellular carcinoma (HCC) is still pessimistic. Therefore, understanding the mechanisms of hepatocellular carcinoma may offer substantial benefits for patients. Our previous research has revealed that Hornerin promoted HCC progression by regulating the AKT signaling pathway. To investigate the upstream regulatory mechanism, the results from RNA Immunoprecipitation and RNA pull-down indicated that the specific region of circIPP2A2 interacted with Hornerin. Additionally, patients with circIPP2A2 upregulation exhibited a poorer survival outcome following surgery compared to the cases with downregulated circIPP2A2. After the structure verification of circIPP2A2, loss-of-function studies using a lentiviral vector revealed that circIPP2A2 downregulation significantly inhibited HCC tumorigenesis and progression both in vitro and in vivo. Mechanistically, the m7G-MeRIP results demonstrated significant enrichment of circIPP2A2. Subsequent studies validated that METTL1 influenced the stability of circIPP2A2 and its binding affinity with Hornerin. Immunoprecipitation and immunofluorescence indicated that circIPP2A2 served as a molecular scaffold to facilitate Hornerin to interact with PI3K. In conclusion, our findings reveal that circIPP2A2, regulated by N7-methylguanosine modification, promotes malignant behaviors in HCC by serving as a molecular scaffold in modulating the Hornerin/PI3K/AKT/GSK3β axis. Targeting circIPP2A2 may be a promising therapeutic strategy for patients with HCC.

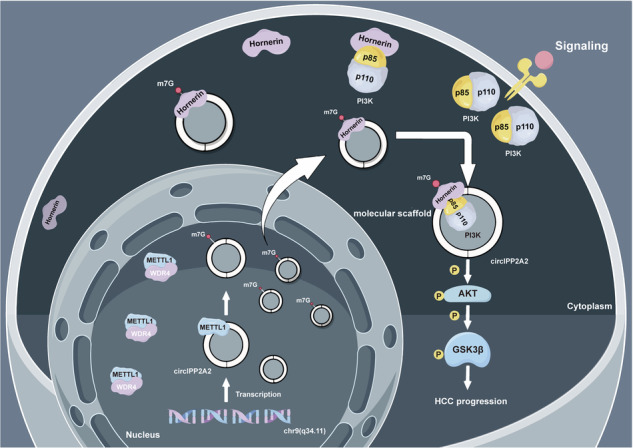

## Introduction

Hepatocellular carcinoma (HCC) is one of the most fetal cancers in the world. In 2020, there were approximately 910,000 new cases and 830,000 deaths worldwide [1]. Half of them occurred in China because of the high prevalence of hepatitis B virus infection [2]. Despite the development of treatment strategies and the emergence of novel therapeutic targets [3], the prognosis of patients with HCC is still not optimistic owing to the shortage of early diagnostic and prognostic monitoring markers for HCC [4]. Therefore, discovering novel biomarkers and understanding the regulatory mechanisms underlying HCC are of great clinical benefit for patients.

Circular RNAs (CircRNAs) are characterized by the connection of a 5’ splice site with the 3’ splice site of a parent pre-mRNA, which is called back-splicing [5]. In past decades, researchers suggested that circRNAs played a critical role in eukaryotes biological processes. CircRNAs function as sponges of miRNAs and proteins and modulators of transcription and translation [6]. Accumulating evidence identified that circRNAs had substantial roles in liver cancer. CircRNA ATCN4 promotes intrahepatic cholangiocarcinoma progression by recruiting YBX1 to initiate FZD7 transcription [7]. CircCPSF6, which is meditated by m6A-modified, promotes HCC progression and metastasis by interacting with PCBP2 to prevent its binding to YAP1 mRNA [8]. Increasing studies suggested that circRNA had a critical involvement in tumorigenesis and development. However, the regulatory mechanisms underlying circRNAs in HCC require further investigation.

N7-methylguanosine (m7G) is one of the most prevalent RNA modifications and is regulated by METTL1–WDR4 complex in humans [9]. METTL1 serves as the catalytic enzyme responsible for the formation of m7G, while WDR4 plays a crucial role in stabilizing the m7G complex [10]. Recent evidence revealed that m7G modification was associated with human biological processes and cancer development. For example, METTL1-meditated m7G modification enhances let-7 miRNA processing by disrupting the secondary structure of primary miRNAs [11], and it may be a new insight into miRNAs structures regulated by RNA modification. In liver cancer, researchers uncovered that METTL1-meditated m7G tRNA modification facilitated intrahepatic cholangiocarcinoma progression by promoting EGFR translation [12]. These findings suggested a correlation between the m7G modification mediated by METTL1 and the occurrence and progression of diseases. However, the mechanism underlying m7G-modified circRNAs still remains to be uncovered.

In a previous study, we reported that Hornerin was an important oncoprotein in HCC. Hornerin overexpression was associated with poor prognosis in HCC patients. In mechanism, Hornerin regulated the AKT signaling pathway to promote HCC progression [13]. Here, this study found that circIPP2A2 had the capability to interact with Hornerin. Notably, circIPP2A2 was found to be upregulated in HCC, and this upregulation was associated with a poor prognosis in patients with HCC. Loss-of-function studies indicated that circIPP2A2 downregulation suppressed tumor growth and metastasis in vitro and in vivo. Mechanism studies identified that the expression and stability of circIPP2A2 are regulated by METTL1-meditated m7G modification. Additionally, circIPP2A2 served as a molecular scaffold to facilitate the interaction between Hornerin and PI3K in HCC. Above all, our data revealed that METTL1-meditated circIPP2A2 promoted malignant behaviors in hepatocellular carcinoma by serving as a scaffold in modulating the Hornerin/PI3K/AKT/GSK3β axis. This study identified that circIPP2A2 may be a potential novel biomarker and a promising therapeutic intervention target for HCC.

## Methods

### Bioinformatic tools

CatRAPID database [14] was used to search circRNAs interacted with Hornerin. CircAtlas 2.0 database [15] was applied to verify the sequence of targeted circRNA. The Cancer Genome Atlas (TCGA) data [16] was used to perform pan-cancer analysis and survival analysis. iRNA-m7G database [17] was used to predict potential m7G modification sites within circIPP2A2.

### Cell lines and clinical tissues

HCC cell lines (HCCLM3, MHCC97H, Huh7) were purchased from the Cell Bank of the Chinese Academy of Sciences (Shanghai, China). HCC cell lines (Hep3B, PLC/PRF5), hepatoblastoma cell line (HepG2), and the normal human liver cell line HL-7702 were obtained from American Type Culture Collection (ATCC). Human 293T cell lines were purchased from ATCC. Short Tandem Repeat profiling was conducted on all cell lines. Cell lines were cultured in high glucose Dulbecco’s modified Eagle medium (DMEM; Gibco, USA) plus 10% fetal bovine serum (FBS; Gibco, USA). Sixteen pairs of HCC and adjacent normal tissues from patients were obtained from Southern Medical University Zhujiang Hospital. A total of 78 HCC tissues and 44 normal liver tissues were obtained from Sun Yat-sen University Cancer Center between January 2005 and December 2010. Informed consent was obtained from all patients. This study was approved by the ethical committee of Zhujiang Hospital and Sun Yat-sen University Cancer Center.

### Total RNA extraction, reverse transcription PCR, and quantitative real-time PCR

TRIzol reagent (Invitrogen, NY, USA) was used to extract RNA from cell lines and tissues according to the standard procedure. Reverse transcription PCR (RT-PCR) was performed using PrimeScript™ RT reagent Kit (Takara, China), SYBR® Green Premix Pro Taq HS qPCR Kit II (AGBIO, China) was used to perform the quantitative real-time PCR (qPCR) according to kit instructions. Glyceraldehyde-3-phosphate dehydrogenase (GAPDH) was used to normalize the expression of RNAs. 2^−ΔΔCt^ method was used to determine the relative expression of RNAs. All primer sequences are listed in Table S1.

### Plasmid construction and cell transfection

The lentiviral vector for circIPP2A2 downregulation (sh-circIPP2A2#1, #2, #3) and the negative control (scramble) were synthesized by Genechem Biotechnology (Shanghai, China). Lentivirus cells were transfected at a multiplicity of infection (MOI) of 15, cells were cultured in DMEM with 3 µg/mL puromycin (Beyotime, China) for HCCLM3 cells and 2 µg/mL puromycin for Hep3B cells for 3 weeks. The small interference RNA (siRNA) targeted METTL1, circIPP2A2, and plasmid overexpressed Hornerin were obtained from Tsingke Biotechnology (Guangzhou, China). Lipofectamine 3000 (Invitrogen, USA) was used to transfect plasmid into cell lines. qPCR and western blotting were used to identify the transfect efficiency. siRNAs and shRNAs sequences are listed in Table S2.

### Western blotting

Proteins from cell lysis and tissues were extracted using Sodium dodecyl sulfate lysis buffer (Beyotime, China) plus proteinase and phosphatase inhibitors (Sigma-Aldrich, NY, USA). The proteins were separated using polyacrylamide gels with varying concentrations of 8%, 10%, or 12% according to their molecular masses. Subsequently, proteins were transferred onto polyvinylidene difluoride membranes (Merck Millipore, Darmstadt, Germany) for further analysis. After that, nonspecific binding was blocked by 5% bovine serum albumin (BSA) or skim milk for 1 h. The membranes were then subjected to incubation with primary antibodies overnight at 4 °C. The next day, 0.05% TBS plus 0.1% Tween-20 was used to wash the primary antibodies three times. And the membranes were incubated with secondary antibodies for 1 h at room temperature. ECL chemiluminescence kits (Invitrogen, NY, USA) were used to detect the protein signals. The antibodies used in the study are listed in Table S3.

### Fluorescence in situ hybridization and Immunofluorescence

Cy3-labeled fluorescent probes targeting circIPP2A2 were purchased from RiboBio (Guangzhou, China). Cells were fixed and permeabilized using a permeabilization buffer (0.1% Triton X-100). Hep3B and HCCLM3 cells were incubated with the probe at 4 °C overnight for hybridization. Washing buffers were used to exclude the unbound probes from samples. DAPI was used for nuclear staining. The probe sequence is listed in Table S2. As for the immunofluorescence assay, cells were fixed and permeabilized according to standard protocol. After that, cells were incubated with primary antibodies at 4 °C overnight. The next day, primary antibodies were removed, and the samples underwent the subsequent step of incubation with secondary antibodies in a light-protected environment. DAPI was employed for nuclear staining. The antibodies used in the study are listed in Table S3. The fluorescence in situ hybridization (FISH) and immunofluorescence (IF) images were captured by a fluorescence microscope (Carl Zeiss Microscopy GmbH, Jena, Germany). The interaction of Hornerin and PI3K was determined by the relative covered area.

### Sanger sequencing

Total RNAs were extracted from HCCLM3 cells and reverse transcribed into cDNA. The PCR products of cDNA amplified by convergent primer were subjected to Sanger sequencing (Tsingke, China). The full-length sequence of circIPP2A2 was obtained and compared to circBase.

### RNase R and actinomycin D treatment

Actinomycin D (2 mg/mL) was used to verify the circular structure of circIPP2A2, and dimethyl sulfoxide (DMSO) (Sigma-Aldrich, NY, USA) was used as the negative control; total RNA was extracted at the indicated time, qPCR was used to detect the expression of circular and linear IPP2A2. Total RNA (3 μg) from HCCLM3 and Hep3B cells were incubated with 2 U/μg RNase R (Geneseed, Guangzhou, China) for 15 min at 37 °C. After treatment, the expression of circular and linear IPP2A2 was measured by qPCR, and DNA agarose gel electrophoresis was used to confirm the results.

### In vitro and in vivo experiments

The standard protocol of in vitro and in vivo studies was performed as described [18]. Briefly, the CCK-8 assay was used to test the proliferation ability change caused by circIPP2A2 downregulation and METTL1 overexpression. Cell density was 1 × 10^3^ cells/well for Hep3B and 2 × 10^3^ cells/well for HCCLM3. The absorbance at 450 nm was measured using Multiskan™ FC (Thermo Scientific, MA, USA). Colony formation was employed to assess the tumorigenesis ability. Cells at a density of 1 × 10^3^ cells/well were seeded in six-well plates and maintained for 14 days. Transwell assays with or without Matrigel (Corning, NY, USA) simulated the invasion and migration process in tumor progression, respectively. Target cells at a density of 5 × 10^4^ were seeded in the upper chamber and incubated for 24 h. A wound healing assay was used to investigate the migration ability at a cell density of 1 × 10^6^ per well. Cells migration across the wound was observed at 0, 48, and 96 h. Above in vitro experiments are presented as the mean ± SD of at least three independent repetitions.

For animal experiments, Hep3B cells at a density of 1 × 10^7^ were resuspended in 150 μL PBS and subcutaneously injected into mice. The width (*W*) and length (*L*) of the tumors were measured every 7 days for 5 weeks. At the end of 5 weeks, the tumors were excised and weighed. HCCLM3 cells at a density of 1 × 10^6^ were intravenous injected into the tail vein of mice to establish the lung metastasis model. After 4 weeks, the fluorescent signal was detected by an In vivo optical imaging system (IVIS).

### RNA immunoprecipitation assay

The RNA immunoprecipitation (RIP) assay was performed using an Imprint RNA Immunoprecipitation kit (Sigma-Aldrich, USA) according to the manufacturer’s protocols. 20 μL of protein A magnetic beads was premixed with 5 μg of anti-Hornerin/METTL1/PI3K antibody or anti-IgG (Sigma-Aldrich) for 1.5 h at room temperature. After that, the mix was added to cell lysates. And incubated at 4 °C overnight. TRIzol reagent was used to extract the total RNA enrichment by Hornerin/METTL1/PI3K and qPCR was used to quantify the expression level.

### Immunoprecipitation assay

Immunoprecipitation (IP) assay was performed according to standard protocol. Proteins from cells were extracted using RIPA buffer (Beyotime, China). The extracted proteins were then incubated with specific antibodies overnight at room temperature with gentle rotation. Subsequently, Protein A agarose was employed to capture the antibody–protein complexes at 4 °C for overnight. The next day, cold PBS was used to wash the nonspecific binding for more than three times. The total proteins were separated using SDS–PAGE and analyzed via western blotting.

### RNA pulls down

Pierce™ Magnetic RNA-Protein Pull-Down Kit (Sigma-Aldrich, USA) was used to conduct RNA pull-down assay according to standard protocol. Initially, Pierce RNA 3´ End Desthiobiotinylation Kit (Thermo Scientific, USA) was used to add a single desthiobiotinylated cytidine bisphosphate to the targeted sequence of the circIPP2A2. The labeled circIPP2A2 was enriched by streptavidin magnetic beads for 30 min at room temperature. 50 μg HCCLM3 and Hep3B cell lysis were added to this mixture. Complex was incubated for 45 min at room temperature. Then, it was eluted after 15 min of incubation at 37 °C with a Biotin Elution Buffer. The enrichment of certain proteins was verified by western blotting.

### m7G-MeRIP assay

A GenSeq® m7G MeRIP kit (GenSeq, China) was used to perform the m7G MeRIP according to instructions. Briefly, total RNAs were extracted using TRIzol reagent (Invitrogen, NY, USA). Subsequently, a 10× Fragmentation Buffer was used for the fragmentation of RNA, with the process carried out at 70 °C for 6 min. After that, a mixture of PC buffer and ethanol was added to the RNA and left at −80 °C overnight for further processing. The next day, 2 μL m7G antibody was pre-mixed with 25 μL PGM magnetic beads for 1.5 h at room temperature with rotation. The RNA fragmentation was then introduced into this mix and underwent incubation at 4 °C overnight. Subsequently, RLT Buffer and ethanol were employed for resuspending and washing the magnetic beads. Random primer was used to perform reverse transcription, and the enrichment of m7G in circIPP2A2 was verified by qPCR assay.

### Statistical analysis

GraphPad Prism 8.0 (La Jolla, CA, USA) and SPSS 21.0 (IBM Corp., NY, USA) were used to perform statistical analysis. Differences between groups were analyzed using Student’s *t*-test or Analysis of Variance. The chi-square test was used to compare categorical variables. The Kaplan–Meier analysis and log-rank test were used to analyze overall survival (OS) and disease-free survival (DFS). Statistical significance was set at *P* < 0.05.

## Results

### CircIPP2A2 interacted with Hornerin to regulate its expression

**Fig. 1:**
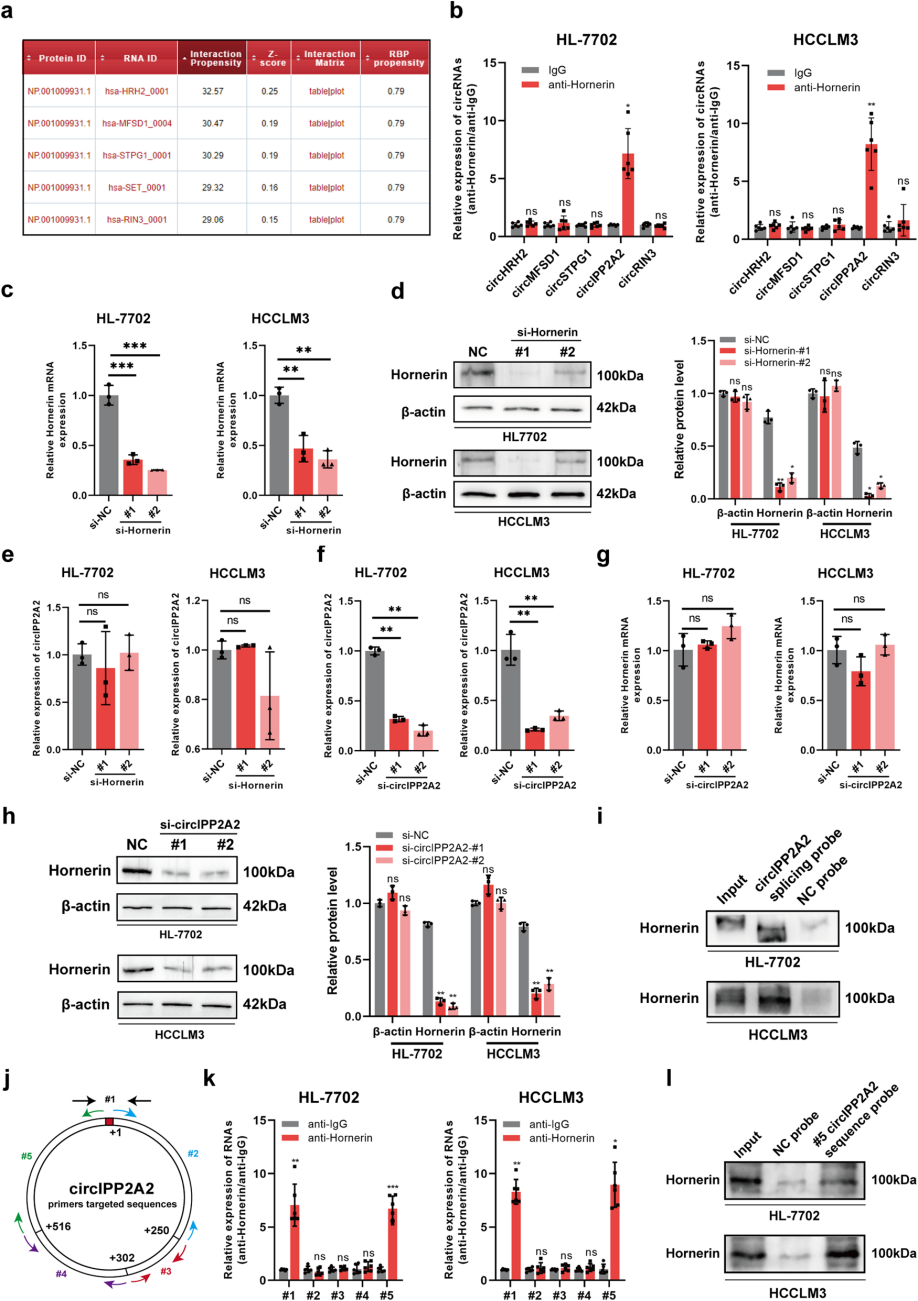
Hornerin had the capability to interact with circIPP2A2. **a** catRAPID results showed the top five circRNAs that interacting with Hornerin. Ranking based on Interaction Propensity. **b** RIP assay using anti-Hornerin antibody in normal liver cells HL-7702 and HCC cells HCCLM3. The binding affinity was assessed using qPCR. **c**, **d** Small interfering RNAs targeted at Hornerin were used to downregulate Hornerin expression. qPCR and western blotting assay were used to verify the successful downregulation. **e** qPCR was used to detect the expression of circIPP2A2 in HL-7702 and HCCLM3. **f** qPCR was employed for the verification of circIPP2A2 silencing. **g**, **h** qPCR and western blotting assay were used to measure the expression of Hornerin in circIPP2A2 downregulated cells. **i** RNA pull-down was performed using Biotinylation labeled circIPP2A2 back splice sequence. Western blotting assay was used to detect the Hornerin signal in HL-7702 and HCCLM3. **j** Schematic diagram of circIPP2A2 primers design. **k** RIP assay was used to unveil the putative sequences with which Hornerin interacted. **l** RNA pull-down using a specific circIPP2A2 probe was employed to validate the RIP result. ns, not significant; *P < 0.05; **P < 0.01; ***P < 0.001.

In the previous study, we identified that Hornerin, a member of the S100 protein family, was overexpressed in patients with hepatocellular carcinoma (HCC), and this upregulation was significantly correlated with poor prognosis [13]. To explore the regulatory mechanism underlying Hornerin-inducing malignant behaviors of HCC, catRAPID database was used to predict the potential interaction between circRNAs and Hornerin. Subsequently, the five most promising circRNAs were selected for RNA immunoprecipitation (RIP) verification in normal liver cells (HL-7702) and one randomly selected HCC cell (HCCLM3) (Fig. 1a and S1). The results indicated that a circRNA derived from the SET nuclear proto-oncogene locus, which is called IPPA22, can bind to the Hornerin protein, thus we named it circIPP2A2 (Fig. 1b). To further explore the mechanism, small interfering RNAs were used to inhibit the Hornerin expression (Fig. 1c, d). qPCR was used to detect the expression level of circIPP2A2 after Hornerin downregulation in HCC cells. The results showed that Hornerin had no effect on regulating circIPP2A2 expression (Fig. 1e). Therefore, we speculated that circIPP2A2 was the upstream regulator of Hornerin. To investigate this hypothesis, circIPP2A2 was silenced using small interfering RNAs (Fig. 1f). However, qPCR result showed that there was no discernible impact detected at the transcriptional level of Hornerin (Fig. 1g). Western blotting results showed that circIPP2A2 downregulation significantly reduced Hornerin expression (Fig. 1h). Further, biotin-labeled circIPP2A2 RNA pull-down was performed and western blotting also verified that circIPP2A2 can bind to Hornerin (Fig. 1i). According to circRAPID database, Hornerin interacted with the +251–302 region of circIPP2A2, so five pairs of primers were designed to detect the specific binding regions (Fig. 1j). However, the results of RIP and RNA pull-down showed that the binding region was at +516–730 (marked at green) which did not align with the initially predicted region (Fig. 1k, l). The aforementioned results indicated that the +516–730 region of circIPP2A2 had the capability to bind with Hornerin and regulate its expression.

### Verification of the expression and structure of circIPP2A2 in HCC

**Fig. 2:**
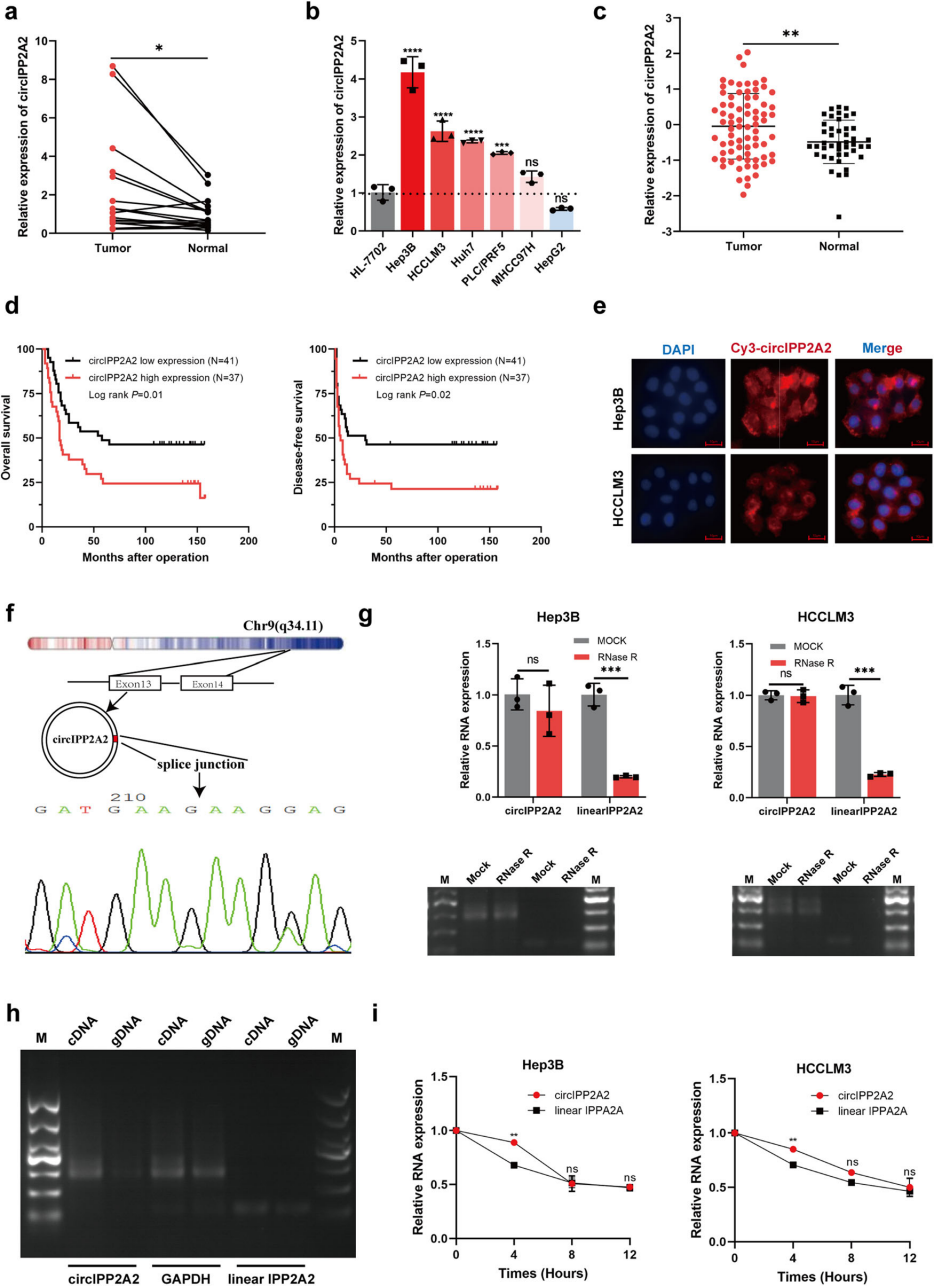
The expression and characteristics of circIPP2A2. **a** Sixteen pairs of HCC and corresponding normal liver tissues were used to detect the circIPP2A2 expression by qPCR. **b** qPCR assay was used to detect the circIPP2A2 expression in HCC cell lines (Hep3B, HCCLM3, Huh7, PLC/PRF5, MHCC97H), hepatoblastoma cell line (HepG2), and normal liver cell lines (HL-7702). **c** qPCR was employed to determine the expression of circIPP2A2 in 78 HCC and 44 normal liver tissues. **d** The expression of circIPP2A2 in 78 HCC tissues was divided into high expression (*N* = 37) and low expression (*N* = 41) group. Kaplan–Meier curves were used to depict the association between circIPP2A2 expression and clinical outcome following operation. **e** FISH assay was used to determine the subcellular location of circIPP2A2 in HCC. **f** Sanger sequencing was used to confirm the back-splicing sequence of circIPP2A2. **g** RNase R digestion assay was conducted to assess the stability of circIPP2A2 in comparison to linear IPP2A2. **h** Convergent primers were applied to amplify GAPDH and linear IPP2A2, while divergent primers were used for circIPP2A2 with cDNA or gDNA as the templates. **i** Actinomycin D treatment impeded the synthesis of linear IPP2A2, while it exhibited no impact on circIPP2A2. ns, not significant; *P < 0.05; **P < 0.01; ***P < 0.001; ****P < 0.0001.

The expression of circIPP2A2 in HCC was detected using 16 pairs of HCC and corresponding normal tissues. qPCR results showed that circIPP2A2 was upregulated in HCC tissues (Fig. 2a). HCC cell lines and human normal liver cell line HL-7702 were used to measure circIPP2A2 expression. It revealed that circIPP2A2 was overexpressed in five HCC cell lines (Hep3B, HCCLM3, Huh7, PLC/PRF5, MHCC97H) and downregulated in hepatoblastoma cell line (HepG2) compared to HL-7702 (Fig. 2b). Further, qPCR was used to determine the expression of circIPP2A2 in 78 HCC tissues and 44 normal liver tissues from Sun Yat-sen University Cancer Center. The result showed that circIPP2A2 was upregulated in HCC tissues compared to normal liver tissues (Fig. 2c). Patients with HCC were divided into two groups: high expression (*N* = 37) and low expression (*N* = 41) according to the median expression of circIPP2A2. Chi-squared test was used to evaluate the clinicopathological features. The results showed that circIPP2A2 high expression was associated with multiple tumor numbers (*P* = 0.02) (Table 1). The Kaplan–Meier curves demonstrated that circIPP2A2 high expression significantly reduced overall survival (*P* = 0.01) and disease-free survival (*P* = 0.02) following surgery compared to the cases with circIPP2A2 low expression (Fig. 2d). The results of multivariate analysis illustrated that vascular invasion and circIPP2A2 high expression were independent risk factors for OS. Additionally, the size of the largest tumor (>5 cm) and the presence of vascular invasion were identified as independent risk factors for DFS (Table 2 and Fig. S2). Above results indicated that circIPP2A2 was upregulated in HCC and associated with poor prognosis.

**Table 1:** Correlations between the expression of circIPP2A2 and HCC pathological characteristics.

Category	Subcategory	Cases	circIPP2A2 expression		*P*-value
			Low (*n* = 41)	High (*n* = 37)	
Gender	Male	66	38 (92.70%)	28 (75.70%)	0.058
	Female	12	3 (7.30%)	9 (24.30%)	
Age (years)	≤60	70	36 (87.80%)	34 (91.90%)	0.416
	>60	8	5 (12.20%)	3 (8.10%)	
HBsAg	Positive	68	34 (82.90%)	34 (91.90%)	0.201
	Negative	10	7 (17.10%)	3 (8.10%)	
AFP (ng/mL)	≤400	47	28 (68.30%)	19 (51.40%)	0.098
	>400	31	13 (31.70%)	18 (48.60%)	
Size of largest tumor (cm)	≤5	33	20 (48.80%)	13 (35.10%)	0.161
	>5	45	21 (52.20%)	24 (64.90%)	
Vascular invasion	Yes	19	7 (17.10%)	12 (32.40%)	0.094
	No	59	34 (82.90%)	25 (67.60%)	
Tumor number	Single	65	38 (92.70%)	27 (73.00%)	**0.020**
	Multiple	13	3 (7.30%)	10 (27.00%)	
Tumor differentiation	Well (I + II)	43	26 (63.40%)	17 (45.90%)	0.093
	Poor (III + IV)	35	15 (36.60%)	20 (54.10%)	
T stage	T0–T2	45	26 (63.40%)	19 (51.40%)	0.198
	T3a–T4	33	15 (36.60%)	18 (48.60%)	
Cirrhosis	Yes	42	20 (48.80%)	22 (59.50%)	0.237
	No	36	21 (51.20%)	15 (40.50%)	

Bold values indicates statistical significant *P* values (*P* < 0.05).

**Table 2:** Multivariate Cox proportional hazards regression model illustrating the prognostic factors for OS and DFS.

Variables	OS			DFS		
	HR	95% CI	*P*	HR	95% CI	*P*
Tumor number (Multiple vs. Single)	1.732	0.862–3.478	0.123	1.282	0.626–2.624	0.497
Size of largest tumor (>5 cm vs. ≤5 cm)	1.375	0.629–3.005	0.425	2.103	1.011–4.373	**0.047**
Vascular invasion (Yes vs. No)	3.538	1.972–6.347	**<0.001**	3.468	1.912–6.292	**<0.001**
circIPP2A2 expression (High vs. Low)	1.815	1.016–3.244	**0.044**	1.378	0.754–2.519	0.297

Bold values indicates statistical significant *P* values (*P* < 0.05).

Next, the feature of circIPP2A2 was verified. The FISH assay using Cy3 labeled circIPP2A2 probe revealed that circIPP2A2 mostly localized in the cytoplasm (Fig. 2e). Sanger sequencing was used to verify the full-length sequence of PCR product amplified by convergent primer targeted circIPP2A2 back-splice junction (Fig. 2f). The expression of linear IPP2A2 was eliminated after RNase R treatment compared to circIPP2A2, which indicated that the circular structure of circIPP2A2 resisted to RNase R digestion (Fig. 2g). Convergent primers were used to amplify linear IPP2A2, and divergent primers were used for circIPP2A2 with cDNA or gDNA as a template (Fig. 2h). The results of Actinomycin D treatment indicated that the synthesis of circIPP2A2 cannot be inhibited by Actinomycin D compare to linear IPP2A2 (Fig. 2i). Our findings confirmed the subcellular localization and the circular structure of circIPP2A2.

### circIPP2A2 downregulation suppressed malignant behaviors of HCC

**Fig. 3:**
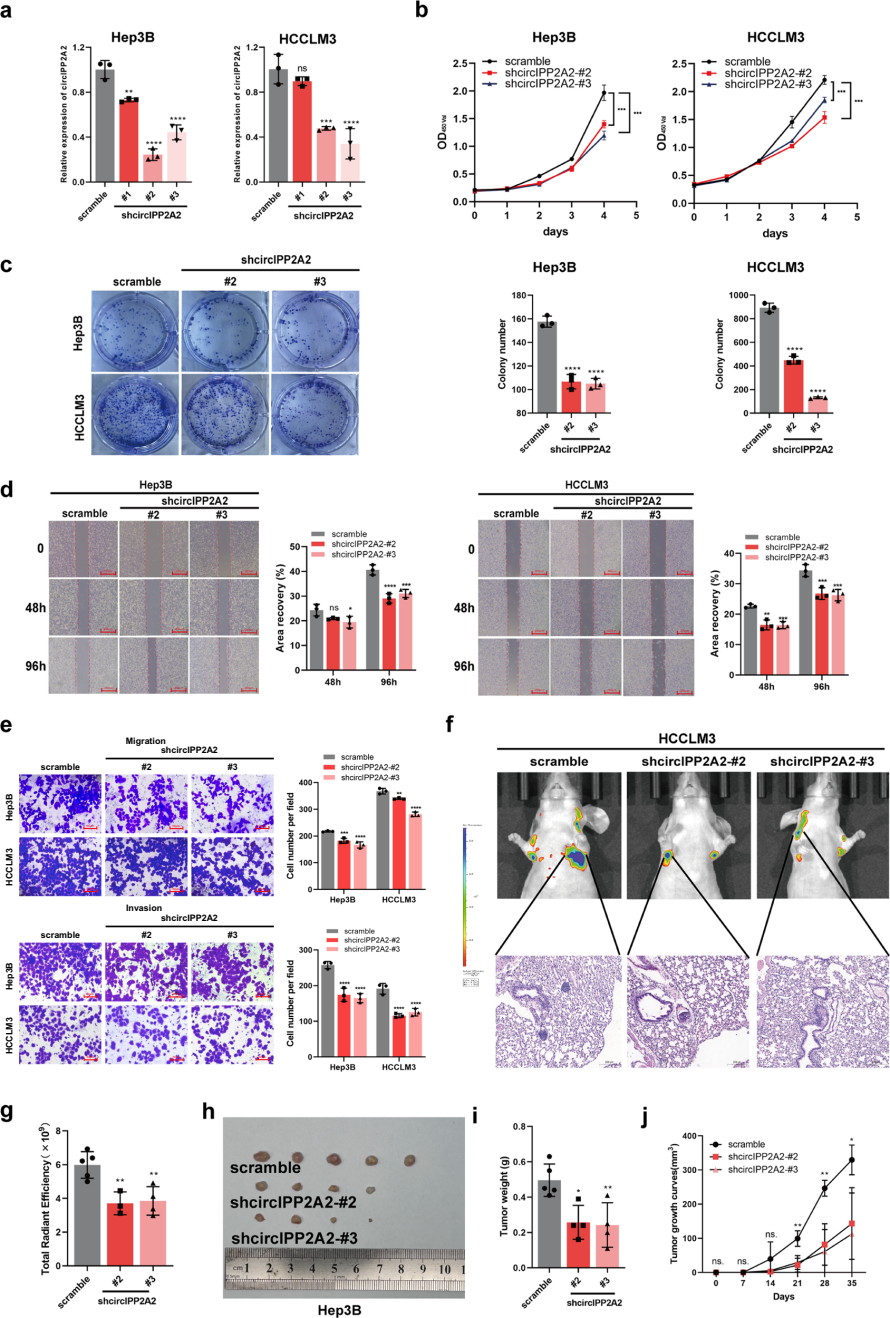
circIPP2A2 downregulation suppressed HCC malignant behaviors in vitro and in vivo. **a** qPCR was used to verify the successful establishment of circIPP2A2 downregulation HCC cell lines. **b** CCK-8 assay was performed to assess the proliferation ability after circIPP2A2 downregulation. **c** Colony formation assay was conducted to evaluate the tumorigenesis change in the context of circIPP2A2 knockdown. **d** Wound healing assay was employed to detect the migration ability after circIPP2A2 downregulation. **e** Transwell chambers with or without pre-coated matrigel were used to simulate the invasion and migration in HCC. **f**, **g** IVIS image showed the metastasis ability change after circIPP2A2 downregulation in nude mice. Fluorescence intensity represented the lung metastasis ability. Metastatic nodes were confirmed by H&E staining. **h** Representative images of xenograft tumors in five nude mice. **i** Changes in tumor volume after circIPP2A2 downregulation. **j** Tumor growth curve illustrated the impact of circIPP2A2 knockdown on proliferation ability. In vitro data are presented as the mean ± SD of at least three independent experiments. ns, not significant; *P < 0.05; **P < 0.01; ***P < 0.001; ****P < 0.0001.

For further investigation, two circIPP2A2 stable knockdown HCC cell lines (Hep3B-shcircIPP2A2#1, #2, #3 and HCCLM3-shcircIPP2A2#1, #2, #3) were established using lentiviral vectors, after Puromycin selection, qPCR was used to confirm the successful construction (Fig. 3a). According to qPCR results, we selected #2 and #3 stable transfected cell lines for subsequent studies. The results of the CCK-8 assay determined that knockdown circIPP2A2 expression attenuated the proliferation abilities in HCC significantly (Fig. 3b). Colony formation results indicated that circIPP2A2 downregulation had the potential to inhibit colony formation abilities in HCC (Fig. 3c). To investigate the metastasis and invasion abilities affected by circIPP2A2, wound healing, and Transwell assays were performed. The results of the wound healing assay implicated that circIPP2A2 downregulation inhibited migration in HCC (Fig. 3d). The results of Transwell assays indicated that knockdown circIPP2A2 expression suppressed both invasion and migration in HCC (Fig. 3e). Above results identified that circIPP2A2 downregulation can inhibit malignant behaviors of HCC in vitro.

Subsequently, a mice model was used to investigate the cancer suppressive effect caused by inhibiting circIPP2A2 expression in vivo. To explore the metastasis ability affected by circIPP2A2 downregulation. The 3-week BALB/c nude mice were used to establish the lung metastasis model through the intravenous injection of HCCLM3 cell lines into the tail vein. The In vivo image system (IVIS) results indicated that silencing circIPP2A2 expression can inhibit lung metastasis in HCC. H&E staining confirmed the lung metastatic node (Fig. 3f, g). A cell derived xenograft (CDX) model was established by injecting Hep3B cell lines subcutaneously. The results showed that circIPP2A2 downregulation inhibited tumor growth and led to a lower tumor burden than that of the scramble group (Fig. 3h–j). Taken together, these results conclusively demonstrated that circIPP2A2 downregulation impeded the malignant behaviors of HCC both in vitro and in vivo.

### CircIPP2A2 was regulated by METTL1-meditated m7G modification

**Fig. 4:**
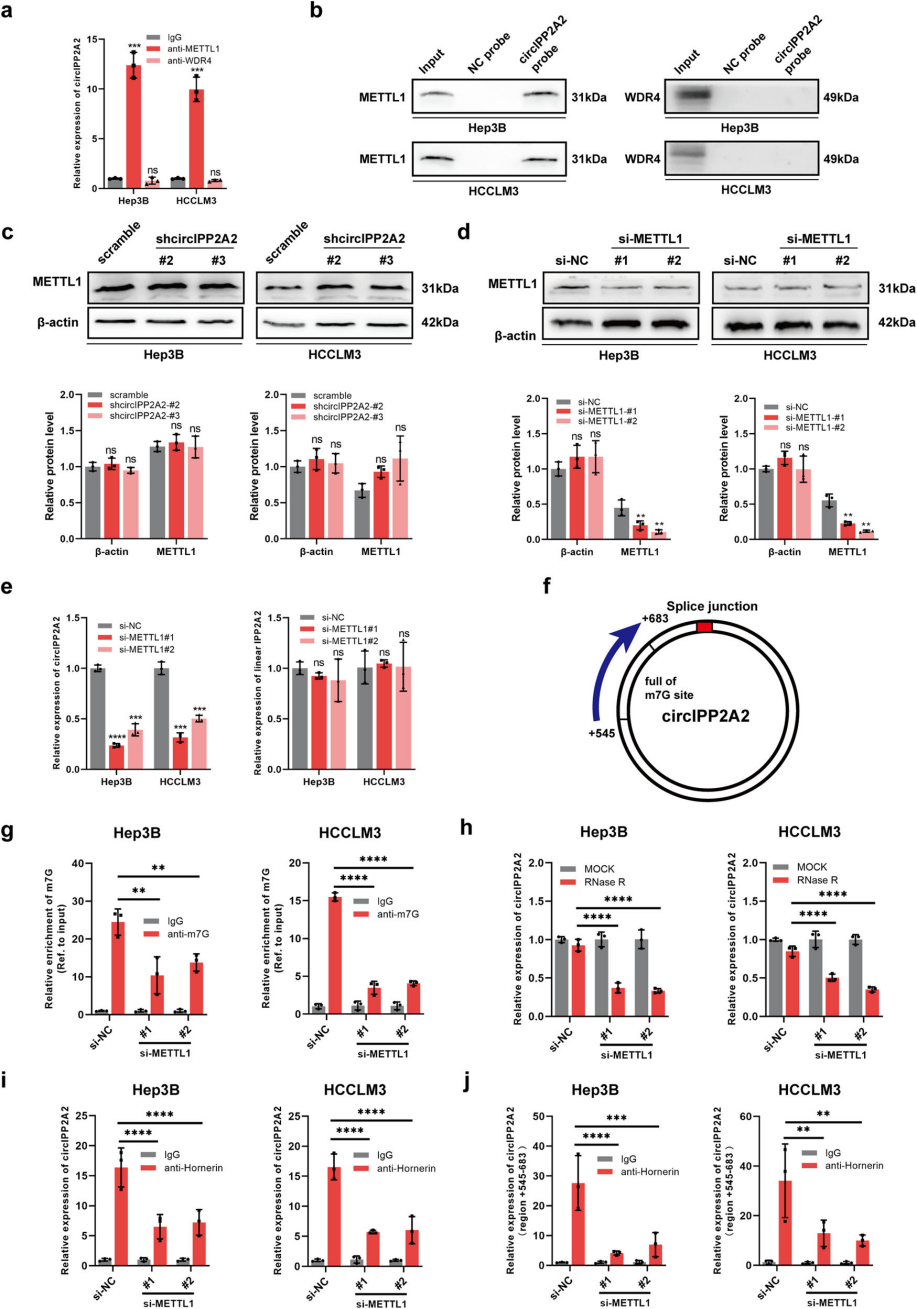
circIPP2A2 was regulated by METTL1. **a** Anti-METTL1 and anti-WDR4 antibodies were used for RIP assay. qPCR was employed to detect the interaction between circIPP2A2 and METTL1 or WDR4. **b** RNA pull-down was performed using Biotinylation labeled circIPP2A2 back splice sequence to capture the METTL1 or WDR4 protein. Western blotting was used to detect the indicated protein signal. **c** METTL1 protein expression after circIPP2A2 downregulation was measured by western blotting. **d** Confirmation of METTL1 silenced by siRNAs. **e** The expression of circIPP2A2 and linear IPP2A2 in the context of METTL1 downregulation. **f** Schematic diagram of prediction m7G site within circIPP2A2. **g** m7G-MeRIP-qPCR was performed to assess the impact of METTL1 on the enrichment of circIPP2A2. **h** RNase R digestion assay was used to measure the stability of circIPP2A2 in the context of METTL1 downregulation. **i**, **j** RIP assay using anti-Hornerin antibody was conducted to investigate the influence of METTL1 on the interaction between Hornerin and circIPP2A2. ns, not significant; *P < 0.05; **P < 0.01; ***P < 0.001; ****P < 0.0001.

In recent studies, N7-methylguanosine was regarded as one of the most extensive RNA modifications. And it has been reported that played a critical role in eucaryote biological possesses and human disease [19]. METTL1-WDR4 complex was the key regulator of m7G modification. METTL1 was the catalytic enzyme of m7G, and WDR4 stabilized the complex [20]. To identify the proteins involved in the m7G modification of circIPP2A2 and their interaction with the RNA molecule, RIP assays were conducted using antibodies against METTL1 and WDR4. Interestingly, the qPCR was used to identify that METTL1 exhibits binding affinity to the sequence of circIPP2A2, instead of WDR4 (Fig. 4a). RNA pull-down assay were used to verify these results (Fig. 4b). Subsequent western blotting and qPCR assays revealed that there was no significant alteration in both mRNA and protein levels of METTL1 in stable circIPP2A2 knockdown cell lines (Fig. 4c and S3a). Intriguingly, silencing METTL1 expression by using siRNAs (Fig. 4d), the qPCR results demonstrated that METTL1 downregulation had the capability to inhibit circIPP2A2 expression, but the linear IPP2A2 expression remained unchanged (Fig. 4e). Therefore, above results determined that METTL1 was the upstream regulator of circIPP2A2.

Considering the pivotal role of m7G modification in cancer development, the iRNA-m7G database, an online bioinformatic tool, was used to predict potential m7G modification sites within circIPP2A2. According to iRNA-m7G database, most of m7G sites in circIPP2A2 were located at +545–683 region (Fig. S3b). m7G-MeRIP assay using anti-m7G antibody was used to verify the m7G modification site in circIPP2A2. And the primers targeted above position were designed for qPCR detection (Fig. 4f). The result of m7G-MeRIP-qPCR revealed that potential m7G-modified region of circIPP2A2 can be captured by anti-m7G, silencing METTL1 expression decreased the m7G enrichment of circIPP2A2 (Fig. 4g). Previously studies revealed that m7G modification had the potential to augment mRNA stability and promote translation. In light of these findings, the stability of circIPP2A2 was tested. qPCR results supported that silencing METTL1 expression rendered circIPP2A2 susceptible to RNase R digestion (Fig. 4h). This suggested that m7G modification enhanced circIPP2A2 stability. Moreover, silencing METTL1 expression results in a notable reduction in the enrichment of Hornerin by circIPP2A2 (Fig. 4i). Take a step further, qPCR was used to detect the binding using the primers targeted m7G site in circIPP2A2. Surprisingly, our results revealed a strong interaction between Hornerin and the m7G-modified region (+545–683) of circIPP2A2 in HCC (Fig. 4j). Above results indicated that circIPP2A2 was regulated by METTL1-meditated m7G modification, and m7G modification within the region of circIPP2A2 plays a crucial role in facilitating the binding of Hornerin, potentially contributing to the development or progression of HCC.

### METTL1 was upregulated in HCC and associated with poor prognosis

**Fig. 5:**
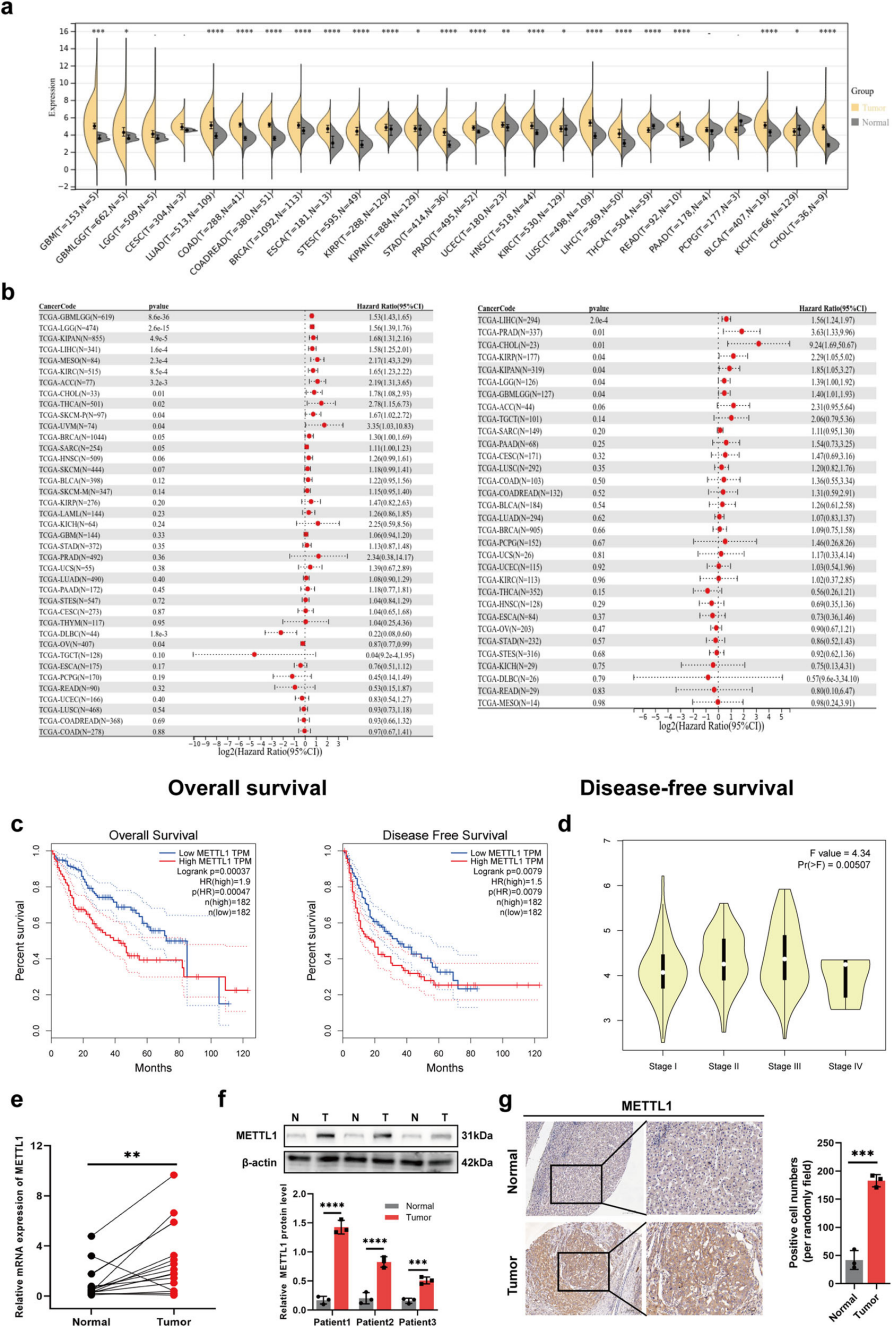
Clinical and survival analysis of METTL1 in various tumors. **a**, **b** A pan-cancer survival analysis was performed to determine the expression of METTL1 in various cancers. **c** The association between METTL1 and clinical outcome was measured using Kaplan–Meier curves in patients with HCC. **d** Violin plot depicted the correlation between METTL1 expression and tumor postoperative pathological grade in HCC. **e** qPCR assay was used to measure the expression of METTL1 in 16 pairs of HCC and corresponding normal liver tissues. **f**, **g** Western blotting and IHC were performed to assess the protein level of METTL1 in HCC and corresponding normal liver tissues. ns, not significant; *P < 0.05; **P < 0.01; ***P < 0.001; ****P < 0.0001.

To explore the biological role of METTL1 in various types of cancer, a pan-cancer analysis was performed to evaluate the expression of METTL1 across different cancer types. The results showed that METTL1 was upregulated in most cancers, including liver cancer (LIHC) and bile duct cancer (CHOL) (Fig. 5a, b). Additionally, survival analysis using Kaplan–Meier curves from the GEPIA database showed that higher METTL1 expression was associated with poorer OS and DFS than lower expression in patients with HCC (Fig. 5c). Furthermore, METTL1 expression was found to be associated with tumor postoperative pathological grade in HCC (Fig. 5d). For further verification, qPCR was used to detect the METTL1 expression in 16 pairs of HCC and corresponding normal liver tissues. The results showed that METTL1 was overexpressed in HCC tissues compared to corresponding normal liver tissues (Fig. 5e). As for protein level, HCC and para-carcinoma tissues were used for western blotting and IHC analysis, the results confirmed that METTL1 was upregulated in HCC (Fig. 5f, g).

**Fig. 6:**
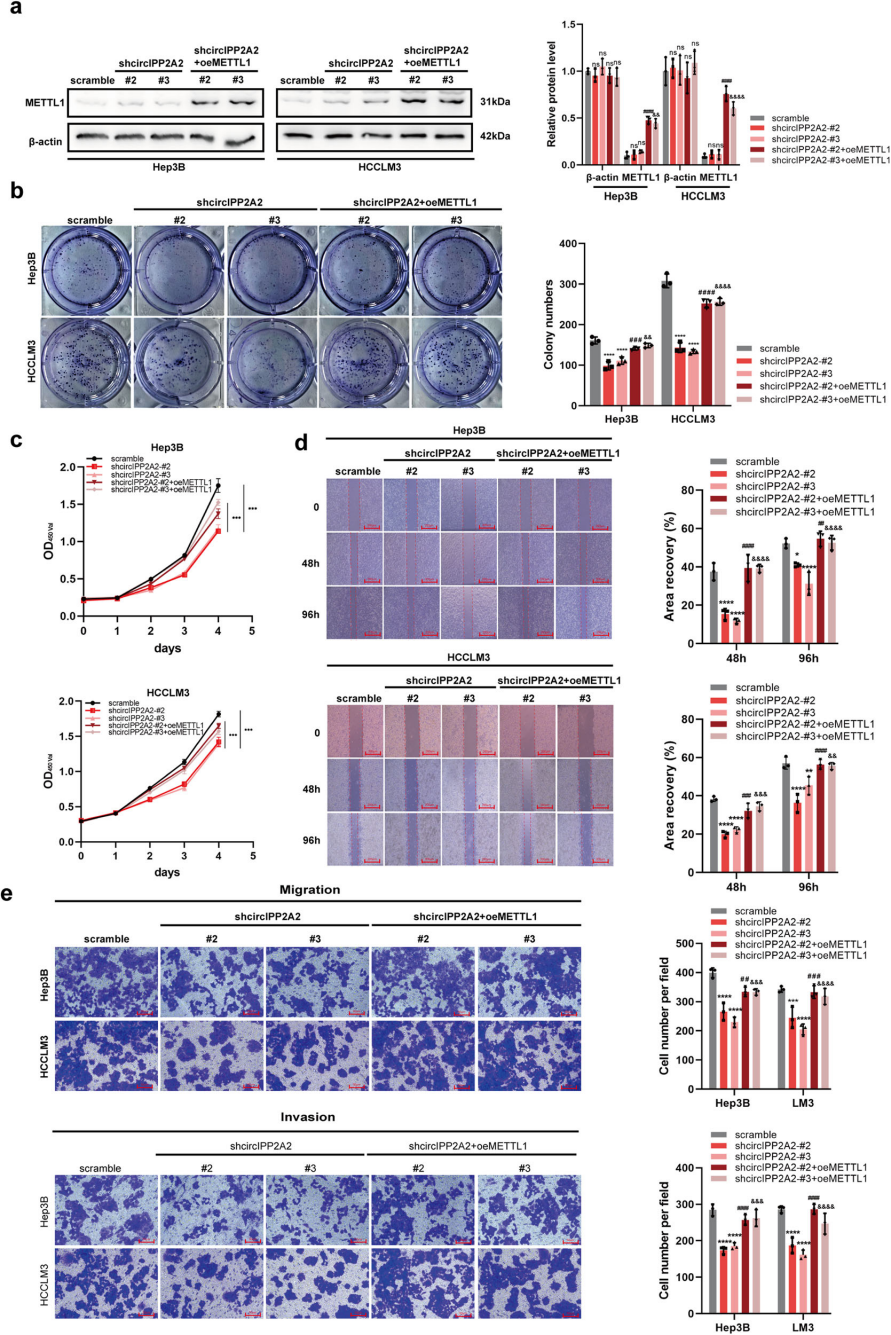
METTL1 overexpression promoted HCC tumorigenesis and metastasis. **a** Western blotting was used to validate the successful overexpression of METTL1 in circIPP2A2 stable knockdown HCC cell lines. **b** Colony formation assay was employed to assess the tumorigenesis ability induced by METTL1 overexpression in the context of circIPP2A2 knockdown. **c** CCK-8 was conducted to detect the impact of METTL1 expression on the proliferation ability. **d** Wound healing assay was performed to measure the migration ability change caused by METTL1 overexpression in circIPP2A2 stable knockdown cells. **e** Transwell assays with or without pre-coated matrigel were used to determine the alterations in migration and invasion induced by METTL1 overexpression, respectively. ns, not significant; */#/& *P* < 0.05; **/##/&& *P* < 0.01; ***/###/&&& *P* < 0.001; ****/####/&&&& *P* < 0.0001.

Further, an overexpression plasmid for METTL1 was employed to explore its biological effects in the context of circIPP2A2 knockdown. Western blotting and qPCR were employed to confirm the successful overexpression of METTL1 (Fig. 6a and S4). The results of colony formation and CCK-8 assays performed in stable silencing circIPP2A2 cell lines confirmed that METTL1 overexpression enhanced the proliferation and colony formation abilities in HCC (Fig. 6b, c). Wound healing and Transwell assays were performed to explore the migration and invasion caused by METTL1 in stable knockdown circIPP2A2 HCC cell lines. The results determined that METTL1 upregulation in the context of circIPP2A2 downregulation rescued the migration and invasion in HCC (Fig. 6d, e). Above results indicated that METTL1, a key protein of N7-methylguanosine modification, was upregulated in patients with HCC. Specifically, the study suggested that METTL1 played a crucial role in the tumorigenesis and progression mediated by circIPP2A2.

### CircIPP2A2 acted as a scaffold to regulate PI3K/AKT/GSK3β signaling pathway

**Fig. 7:**
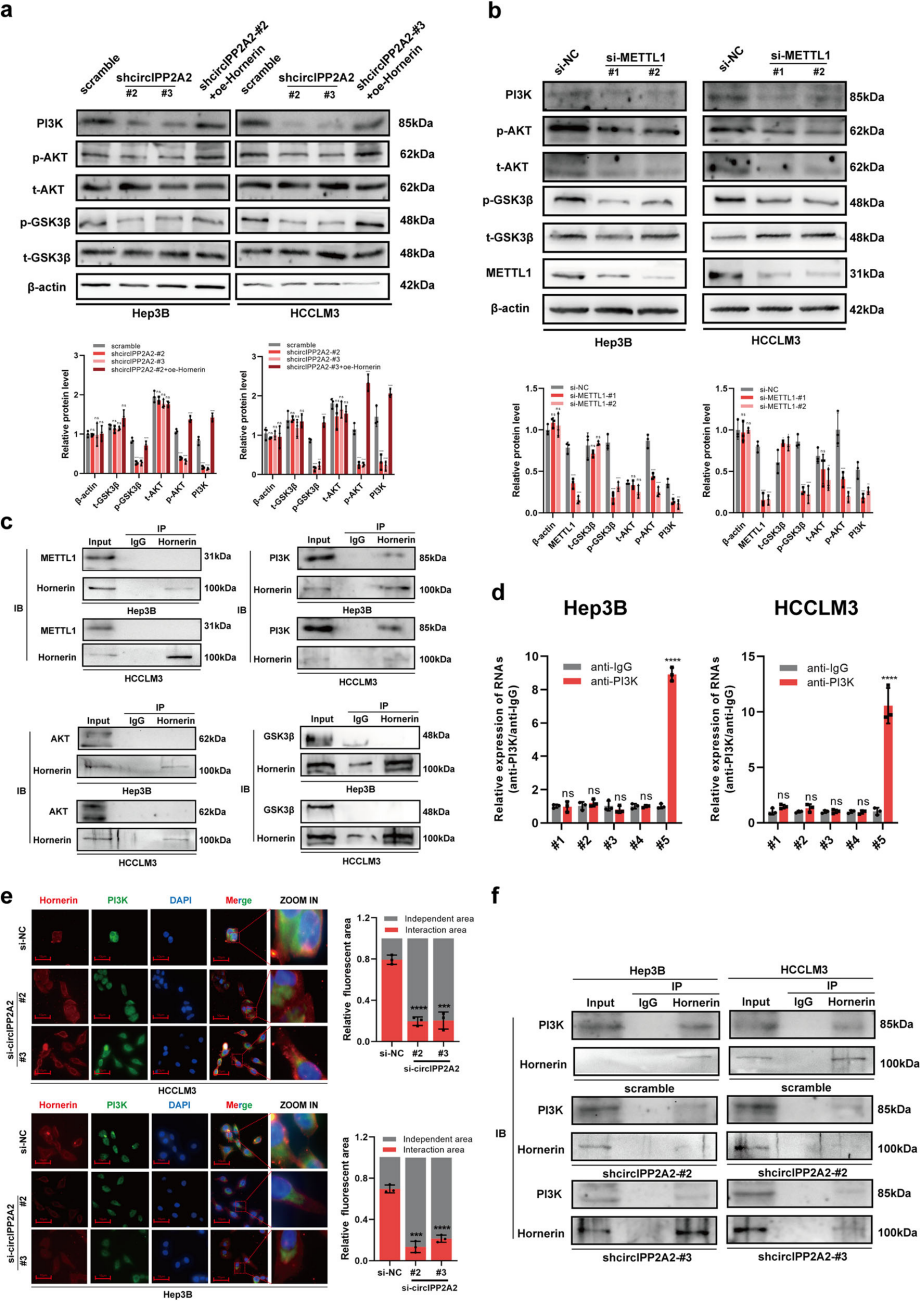
circIPP2A2 served as a scaffold in modulating the Hornerin/PI3K/AKT/GSK3β axis. **a** Western blotting was used to detect the key protein level of the PI3K/AKT/GSK3β signaling pathway in circIPP2A2 stable knockdown and Hornerin overexpression. **b** The activity of the PI3K/AKT/GSK3β signaling pathway was measured by western blotting after METTL1 downregulation. **c** IP assay was performed to assess the binding affinity of Hornerin with METTL1, PI3K, AKT, and GSK3β. **d** RIP assay was employed to measure the interaction between PI3K and the specific sequences of circIPP2A2. **e** Immunofluorescence using anti-Hornerin (red) and anti-PI3K (green) antibodies were conducted to evaluate the alteration in affinity between Hornerin and PI3K upon silencing of circIPP2A2. **f** IP assay was used to observe alterations in the interaction between PI3K and Hornerin within circIPP2A2 knockdown HCC cells. ns, not significant; *P < 0.05; **P < 0.01; ***P < 0.001; ****P < 0.0001.

In the previous article, we confirmed that Hornerin can regulate the AKT signaling pathway [13]. As an upstream regulator of Hornerin, circIPP2A2 may also regulate the PI3K/AKT/GSK3β signaling pathway. To confirm the above hypothesis, western blotting was conducted to detect the protein level of PI3K, AKT, Phospho-AKT, GSK3β, and Phospho-GSK3β. The results indicated that circIPP2A2 downregulation inhibited the PI3K/AKT/GSK3β signaling pathway. Further, Hornerin was upregulated using plasmid in stable knockdown circIPP2A2 cell lines. The results revealed that Hornerin overexpression rescued the inhibition effect caused by circIPP2A2 downregulation in HCC cell lines (Fig. 7a). This study has identified that circIPP2A2 was regulated by METTL1-meditated m7G modification previously. Hence, we postulated that METTL1 might also exert its regulatory influence on the PI3K/AKT/GSK3β signaling pathway. To explore this hypothesis, western blotting assays were performed to detect the expression of the key proteins in this pathway after inhibiting METTL1 expression by siRNAs. The results showed that silencing METTL1 expression also inhibited the PI3K/AKT/GSK3β signaling pathway (Fig. 7b). Furthermore, the IP assay indicated that Hornerin exhibited an interaction with PI3K rather than METTL1, AKT, or GSK3β (Fig. 7c). The results of RIP assay confirmed that PI3K had the capability to bind with circIPP2A2, notably at the shared binding region with Hornerin (+545–683) (Fig. 7d). In the Immunofluorescence results, the co-localization of Hornerin and PI3K within the cytoplasm provided additional evidence supporting the notion that these two proteins indeed interacted with each other. Intriguingly, when circIPP2A2 was inhibited by siRNAs, the interaction between PI3K and Hornerin exhibited a noticeable decrease (Fig. 7e). Regarding the previous findings indicated an interaction between circIPP2A2 and Hornerin. We suggested that circIPP2A2 may acted as an “scaffold” to facilitate the Hornerin interacted with PI3K. IP assay was performed using circIPP2A2 stable knockdown HCC cell lysis. The results determined that inhibiting circIPP2A2 expression blocked the interaction between Hornerin and PI3K in HCC (Fig. 7f). Taken together, our results indicated that circIPP2A2 acted as a scaffold to facilitate the interaction between Hornerin and PI3K to regulate the AKT/GSK3β signaling pathway.

## Discussion

Non-coding RNAs (ncRNAs) were once regarded as by-products of the gene transcription process. And only a few ncRNAs have been identified in human diseases [21]. With the rapid development of high-throughput sequencing and bioinformatic tools, millions of crucial non-coding RNAs (ncRNAs) have been identified over the past decade. These ncRNAs played integral roles in human biological processes and contributed to the progression of various diseases [22]. CircRNAs that lack the 5’ cap and 3’ poly-A tail were regarded as a special type of ncRNAs [23]. In our recent study, circDHPR was identified as a novel circular RNA that was aberrantly expressed in HCC. circDHPR downregulation was associated with poor prognosis in patients with HCC. In mechanism, circDHPR was found to inhibit liver cancer tumorigenesis and progression by regulating the RASGEF1B/RAS/MAPK signaling pathway [18]. Recent studies have shown that circRNAs play a pivotal role in tumorigenesis and cancer development, including breast [24], bladder [25], and lung cancers [26]. The underlying mechanism of circRNAs meditated HCC malignant behaviors remains to be further investigated.

Hornerin was first reported as a novel member of the “fused gene”-type cornified envelope precursor protein family in 2001 [27]. In recent years, an increasing number of researchers identified that Hornerin regulated pathophysiological processes of tumor, such as breast cancer [28] and pancreatic ductal carcinoma [29]. Previously, we have reported that Hornerin, a member of the S100 protein family, was overexpressed in HCC. Hornerin downregulation suppressed tumorigenesis and metastasis in HCC [13]. For finding the Hornerin-specific bind region with circIPP2A2, the catRAPID database was used to predict the possible site where circIPP2A2 binds to Hornerin. Interestingly, the predicted region (+251–302) was not in line with our results of RIP and RNA pull-down assays. Only the qPCR using primers targeted +516–730 region, and back-splicing sequence can be enriched by anti-Hornerin antibody. The RNA pull-down probe using +516–730 region sequence confirmed the results of the RIP assay. Therefore, we determined that Hornerin possessed the aptitude to establish an interaction with the specific region of circIPP2A2. Further studies were employed to confirm that circIPP2A2 was upregulated in HCC and was associated with poor prognosis in patients with HCC. After verifying the feature of circIPP2A2, loss-of-function studies identified that circIPP2A2 downregulation inhibited HCC proliferation and metastasis in vitro and in vivo.

RNA epigenetic modification has been found in all domains of life, demonstrating their pervasive presence in biological systems [30]. Previous evidence had identified that modifications such as N6-methyladenosine (m6A), N5-methylcytosine (m5C), N4-acetylcytidine (ac4C) regulated RNA stability and translational efficacy [31]. In recently, m7G modification attracted the researcher’s attention. However, research about m7G modification in circRNAs has yielded no significant findings so far. Here, we used iRNA-m7G database to find the m7G site in circIPP2A2. The results showed that the +545–683 region of circIPP2A2 contained the most abundant m7G site. Thus, the findings from RIP and RNA pull-down assays revealed that METTL1, rather than WDR4, was identified as the interacting partner with circIPP2A2. qPCR results identified that silencing METTL1 expression downregulated the expression of circIPP2A2, but knockdown circIPP2A2 had no effect on METTL1 expression. Furthermore, the m7G-MeRIP-qPCR using primers targeting the +545–683 region of the circIPP2A2 sequence, demonstrated that this specific region was significantly enriched by the anti-m7G antibody. Additionally, the reduction of METTL1 expression led to a noticeable decrease in the enrichment levels of circIPP2A2. Thus, the METTL1-meditated m7G modification may be the upstream regulatory mechanism of circIPP2A2. It was reported that m7G modification played a pivotal role in the regulation of mRNA stability [32]. Therefore, an RNase R digestion assay was conducted to detect the impact of METTL1 expression interference on the stability of circIPP2A2. The findings revealed that the m7G modification mediated by METTL1 significantly augmented the stability of circIPP2A2. Interestingly, the +516–730 region of circIPP2A2 was identified as binding to Hornerin, encompassing the m7G-enriched region +545–683. Experiments were performed to verify that m7G modification indeed possessed the capability to augment the interaction between circIPP2A2 and Hornerin. Nevertheless, the precise binding position within the +516–730 region of circIPP2A2 and its interaction with Hornerin necessitated further in-depth investigation, especially at the single-base level. Further, METTL1 overexpression rescued the tumorigenesis and metastasis in the context of circIPP2A2 downregulation in HCC. The above results served as a supplement to the m7G modification in regulating human biological processes.

There were ample studies that have determined that PI3K is a vital oncogene [33]. And the downstream target AKT was reported to be upregulated in several types of cancers. PI3K/AKT signaling pathway was regarded as a key promoter in cancer cell malignant behaviors [34]. In a previous study, we verified that Hornerin regulated the AKT signaling pathway to promote HCC progression. Given that circIPP2A2 interacted with Hornerin, it was noteworthy that this interaction could be modulated by m7G modification. We came up with a presumption that circIPP2A2 also promoted the PI3K/AKT signaling pathway in HCC. Western blotting confirmed that circIPP2A2 downregulation suppressed the PI3K/AKT/GSK3β signaling pathway significantly. Further, the binding interaction between Hornerin and PI3K was verified by IP assay. Additionally, circIPP2A2 served as a scaffold to facilitate the binding of Hornerin to PI3K.

## Conclusions

This study was built upon our prior research that Hornerin regulated the AKT signaling pathway in hepatocellular carcinoma. Our finding suggested that circIPP2A2, regulated by METTL1-meditated m7G modification, acted as a scaffold to facilitate the interaction between Hornerin and PI3K, thereby regulating the AKT/GSK3β signaling pathway. CircIPP2A2 may serve as a cancer biomarker for postoperation monitoring and therapeutical intervention for patients with hepatocellular carcinoma.

## Supplementary information

SUPPLEMENTAL MATERIAL

wb original data

## Data Availability

All data generated or analyzed during this study are included in this published article.
