# Supplementary material for: A N7-methylguanosine modified circular RNA, circIPP2A2, promotes malignant behaviors in hepatocellular carcinoma by serving as a scaffold in modulating the Hornerin/PI3K/AKT/GSK3β axis
Source: Cell Death Dis. 2024 Nov 30;15(11):868. doi: 10.1038/s41419-024-07248-7 (PMC11608253; doi:10.1038/s41419-024-07248-7)
Supplement: Supplementary file 1 — SUPPLEMENTAL MATERIAL [file 41419_2024_7248_MOESM1_ESM.docx]

**Table S1. Sequences of qRT-PCR primers used in this study.**

| **Gene** | **Forward primer (5’-3’)** | **Reverse primer (5’-3’)** |
| --- | --- | --- |
| circHRH2 | CCAGGTGTGGAGTGGGACAG | TGATGGAGTCAAGTACTGGGGA |
| circMFSD1 | TGATACTGGATTCTCGGGGGT | CCAAATTCCATCAGCCAAAAAGC |
| circSTPG1 | AATGAACGCACTGGCAAACA | GCAAAATTCTGTGACGTGTTCC |
| cirRIN3 | TGGTGCTCTGTGTCCACTTT | TCCTCTCCTTTGCCAACAACT |
| Liner IPP2A2 | AGATGCTCACTAAGTTCGGAAAAA | CTTCATCTTCCTCCCCAAGCA |
| circIPP2A2-#1 | AATCTGGAAAGAAAAAGAACAGCA | CAAAATCTCCTCACTGGCTTGT |
| circIPP2A2-#2 | TGAACAAGCCAGTGAGGAGA | ACACTTGTGGATGGTTGACA |
| circIPP2A2-#3 | AGAGGTCAGAATTGATCGCCA | ACTGTCACTATCTTTCATCAAGCA |
| circIPP2A2-#4 | TTGCTTGATGAAAGATAGTGACAG | CAACAGGCACACACTAACAACA |
| circIPP2A2-#5 | AATTTTCTGGCCTAGTGTCTGC | TCGGTGGACTTCGAAGATGG |
| Hornerin | AGCTGTTTCTGTCTCTACCCT | CAGTGATGACGCCTTGTAGGA |
| METTL1 | TCTTCGCTCCACTCACTCAAAA | TTTGTCAGCTAACAGGCCAC |
| GAPDH | CACCCACTCCTCCACCTTTG | CCACCACCCTGTTGCTGTAG |

**Table S2. Sequences of RNA FISH and siRNAs/shRNAs targeted sequence**

| **Gene** | **Sequence (5’-3’)** |
| --- | --- |
| shcircIPP2A2-#1 | CCGGGGGAAAAGATATACAGCTACTCGAGTAGCTGTATATCTTTTCCCTTTTTG |
| shcircIPP2A2-#2 | CCGGGAGCAATTGCCTTTAGTTACTCGAGTAACTAAAGGCAATTGCTCTTTTTG |
| shcircIPP2A2-#3 | CCGGCTGTTTAGGAGAAGCTATACTCGAGTATAGCTTCTCCTAAACAGTTTTTG |
| shcircIPP2A2-scramble | CCGGTTCTCCGAACGTGTCACGTTTCAAGAGAACGTGACACGTTCGGAGAATTTTTG |
| sicircIPP2A2-NC | TTCTCCGAACGTGTCACGT |
| sicircIPP2A2-#1 | GAGCAATTGCCTTTAGTTA |
| sicircIPP2A2-#2 | CTGTTTAGGAGAAGCTATA |
| si-Hornerin-NC | CAATGGTGACTCTCGAATGTCGAAA |
| si-Hornerin-#1 | CAAGCTGGTTCAGGCTCGTAATAAA |
| si-Hornerin-#2 | CCAAGAGGAACAAGAAGAAACTGAA |
| si-METTL1-NC | TGTACAGATCTACGGCTCCAGGACT |
| si-METTL1-#1 | TGTGGGACATCTAGGCACCTCAACT |
| si-METTL1-#2 | GGATGTGCACTCATTTCGA |
| Cy3-circIPP2A2 | **Cy3-**GAAAUCUGGAAAGAAAAAGAACAGC |

**Table S3. Antibodies used in Western blotting and immunohistochemistry**

| **Antibody** | **Company** | **Cat No.** |
| --- | --- | --- |
| METTL1 | Proteintech | 14994-1-AP |
| β-actin | Cell Signaling | A4444 |
| Hornerin | Novus Biologicals | NBP1-80807 |
| WDR4 | Abcam | ab169526 |
| PI3K | Proteintech | 60225-1-Ig |
| AKT | Proteintech | 10176-2-AP |
| p-AKT | Proteintech | 66444-1-Ig |
| GSK3β | Proteintech | 22104-1-AP |
| p-GSE3β | Abcam | ab75745 |
| m7G | MBL | RN017M |
| CoraLite594 – conjugated Goat Anti-Rabbit IgG(H+L) | Proteintech | SA00013-4 |
| CoraLite488-conjugated Goat Anti-Mouse IgG(H+L) | Proteintech | SA00013-1 |
| HRP-labeled Goat  Anti-Rabbit IgG(H+L) | Beyotime | A0208 |
| HRP-labeled Goat  Anti-mouse IgG(H+L) | Beyotime | A0216 |
| HRP-labeled Goat  Anti-rat IgG(H+L) | Beyotime | A0192 |


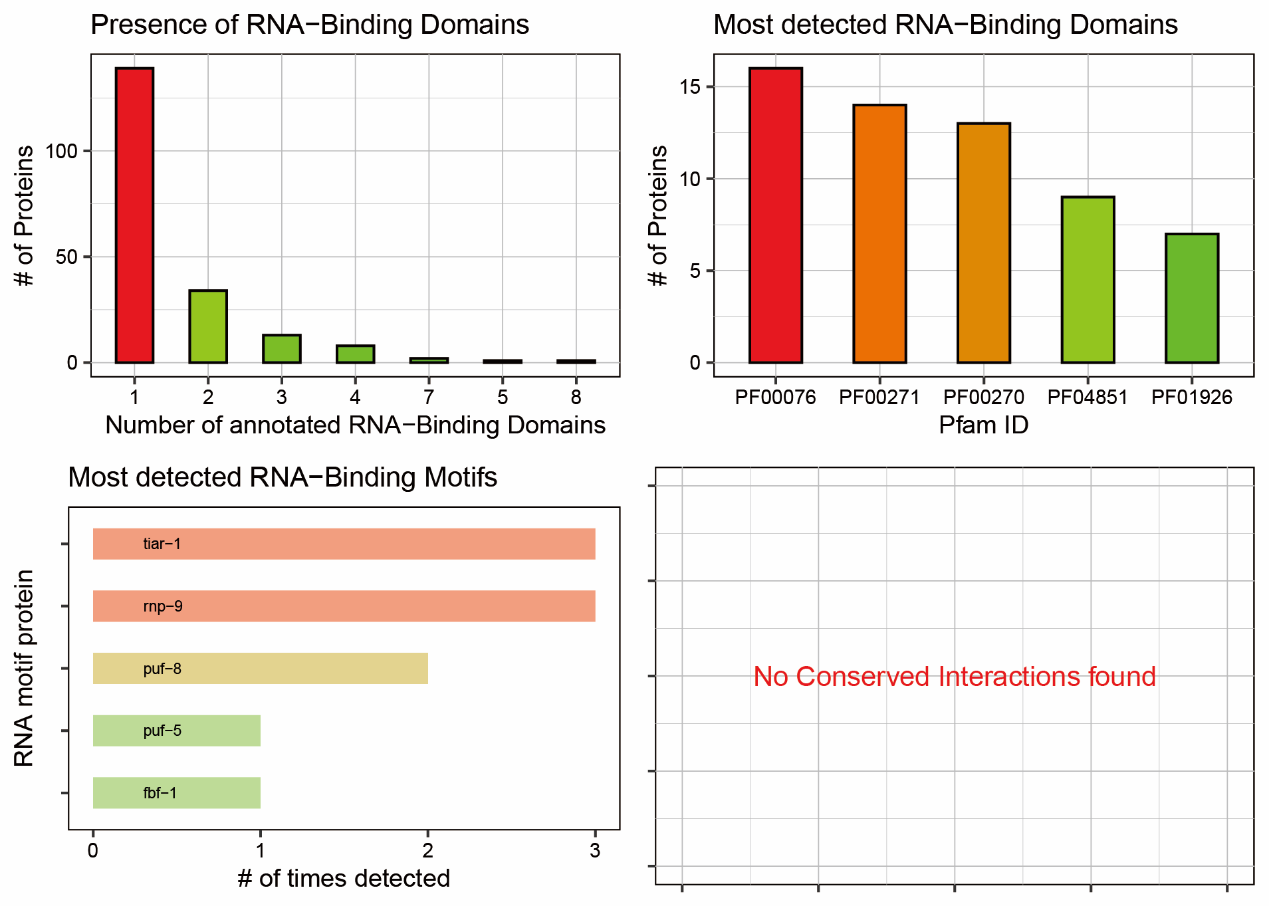


Fig. S1. The RNA binding domains and motifs of Hornerin protein were predicted by catRAPID database.


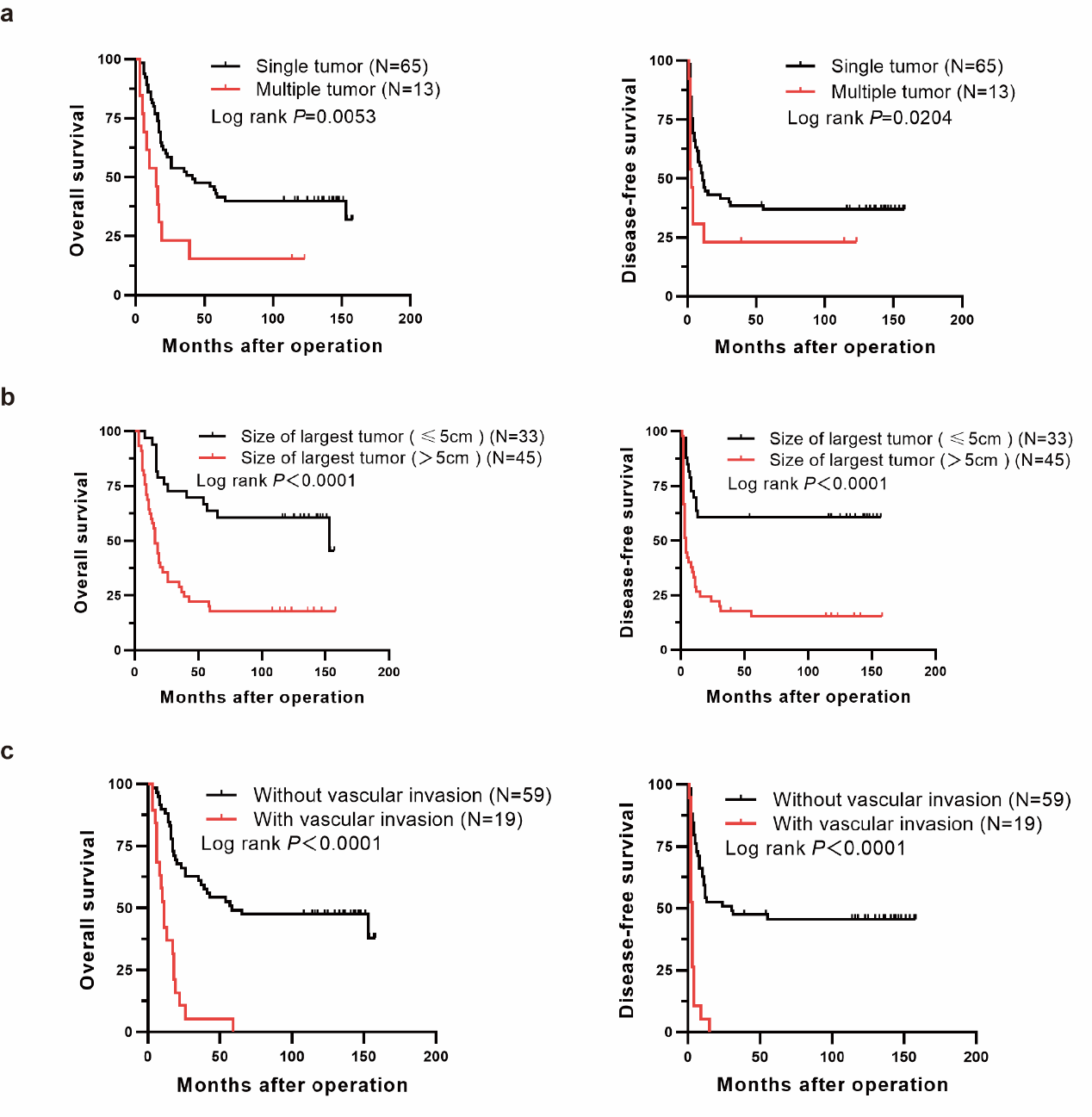


Fig. S2. Kaplan-Meier curves were used to analyze the correlation of pathological characteristics with OS and DFS.


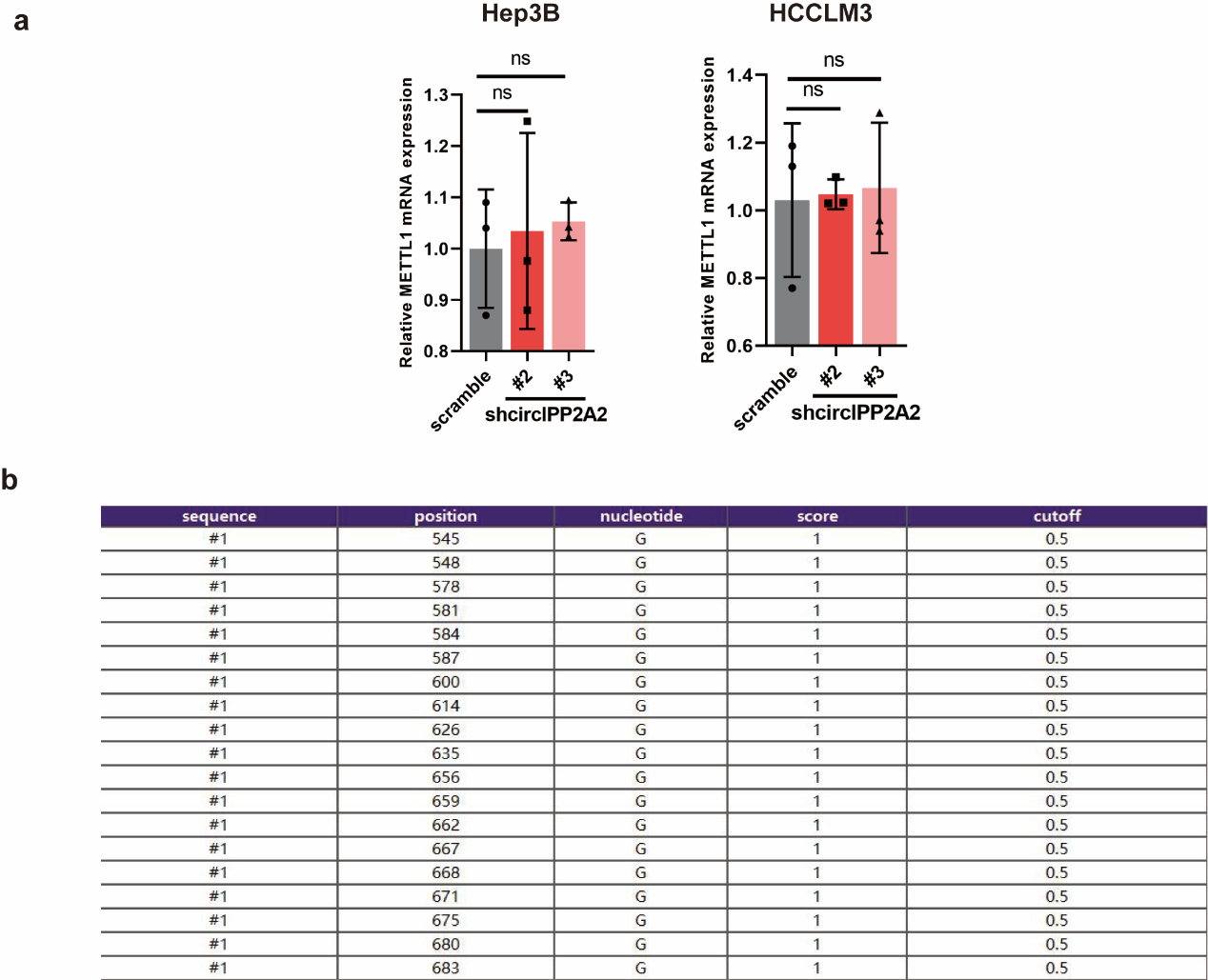


Fig. S3.

a. qPCR was used to detect the expression of METTL1 in circIPP2A2 stable knockdown HCC cells. b. The m7G modification site in circIPP2A2 was predicted by iRNA-m7G database.


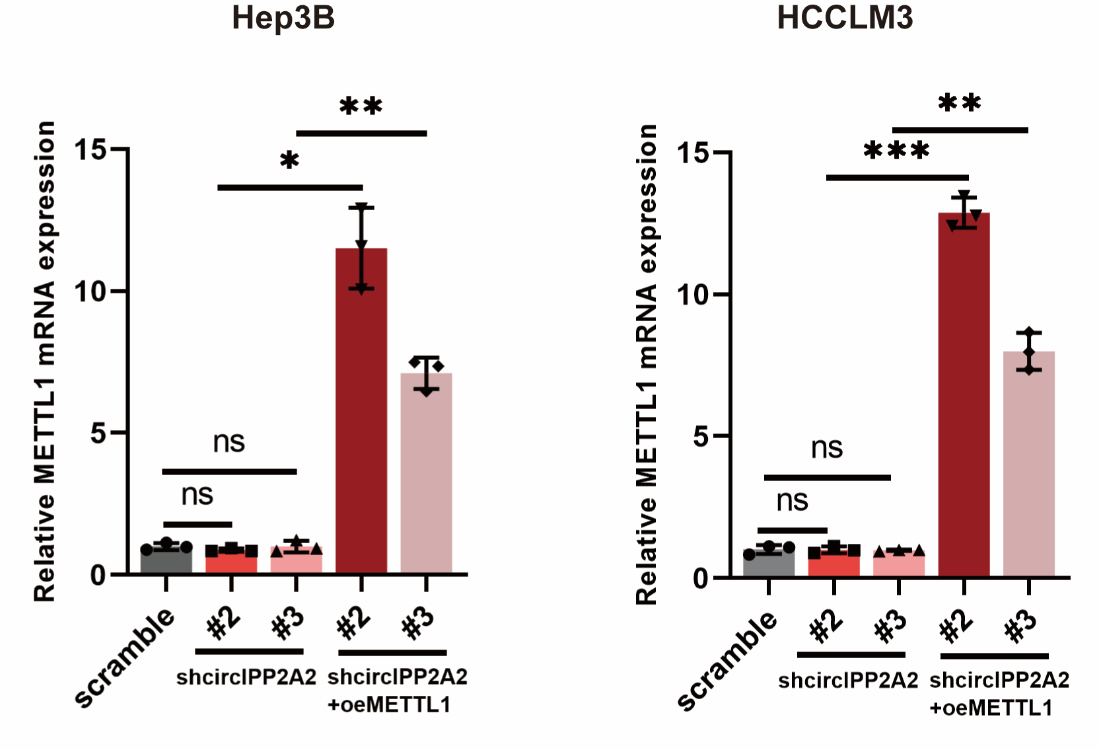


Fig. S4. qPCR was employed to verify the overexpression of METTL1 at the context of circIPP2A2 downregulation in HCC cells.
