# Supplementary figures and images for: A N7-methylguanosine modified circular RNA, circIPP2A2, promotes malignant behaviors in hepatocellular carcinoma by serving as a scaffold in modulating the Hornerin/PI3K/AKT/GSK3β axis
Source: Cell Death Dis. 2024 Nov 30;15(11):868. doi: 10.1038/s41419-024-07248-7 (PMC11608253; doi:10.1038/s41419-024-07248-7)

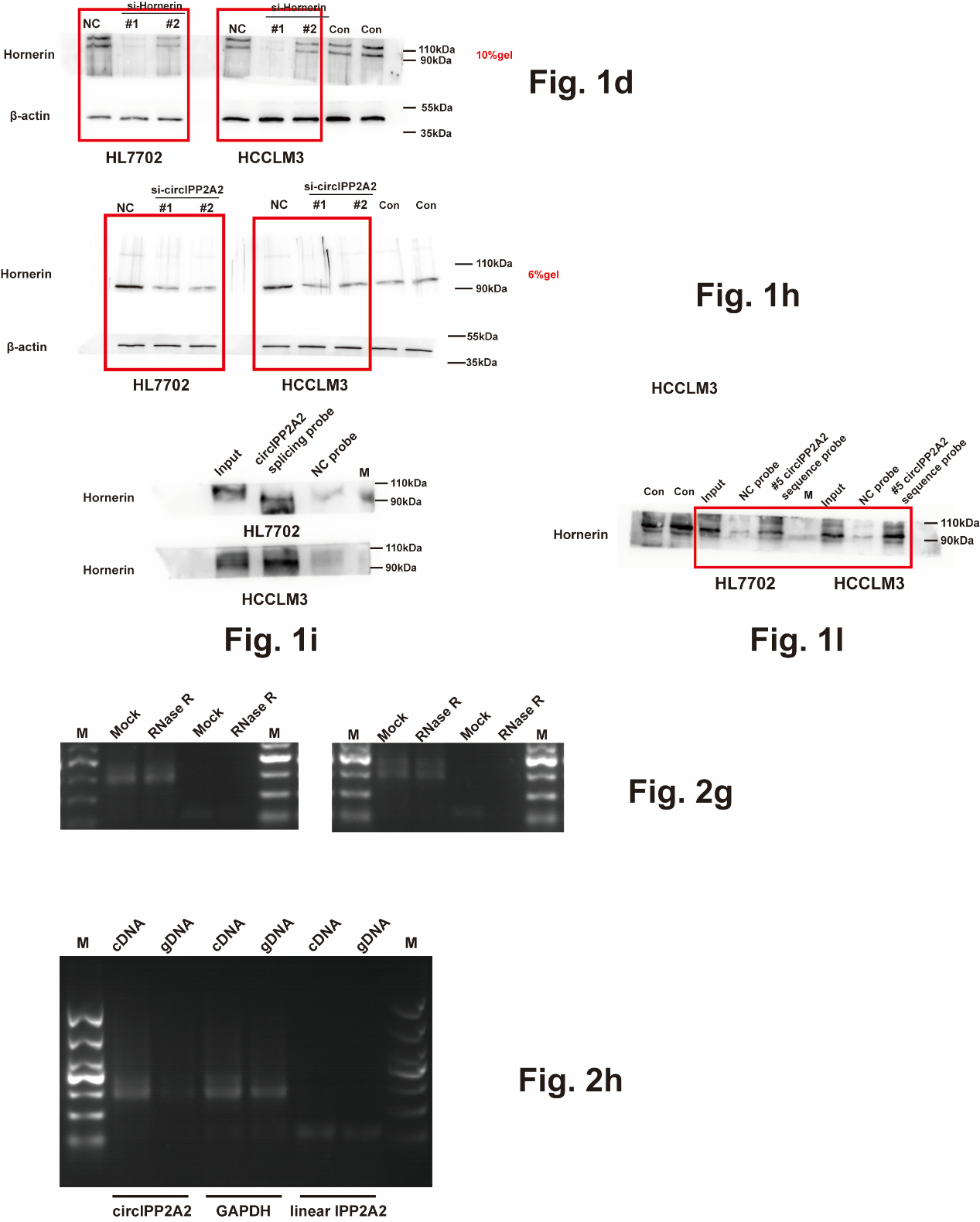


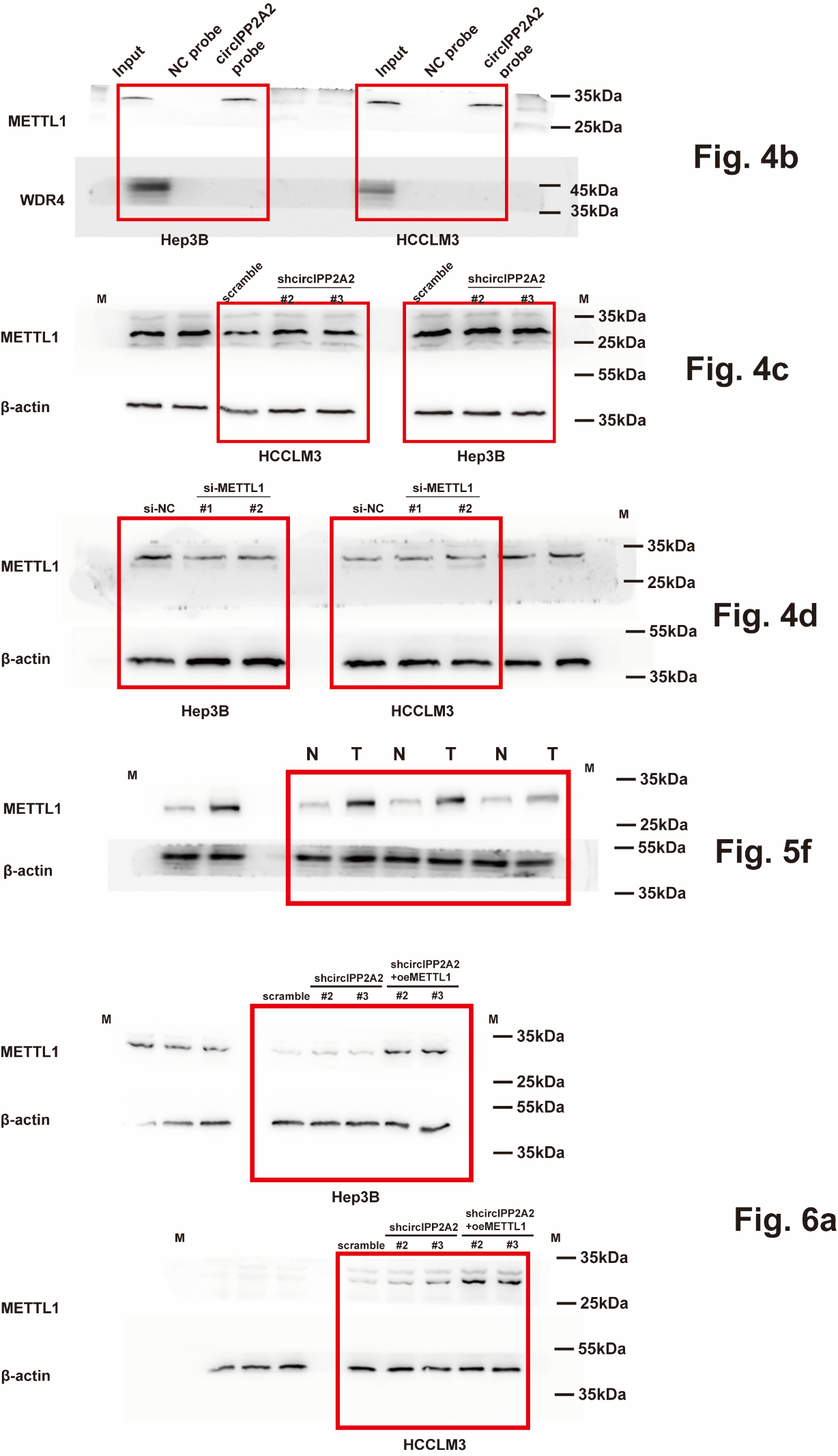


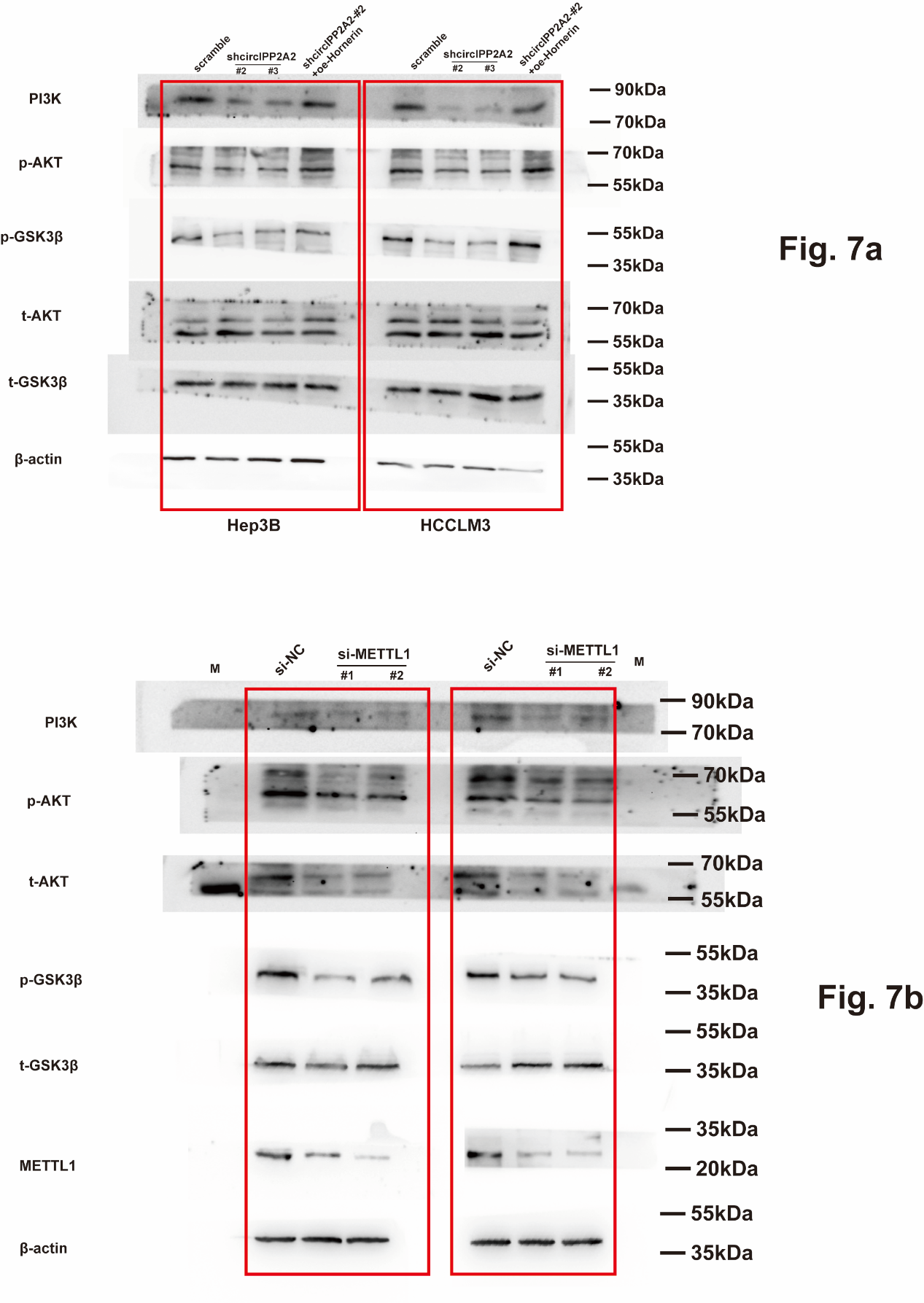


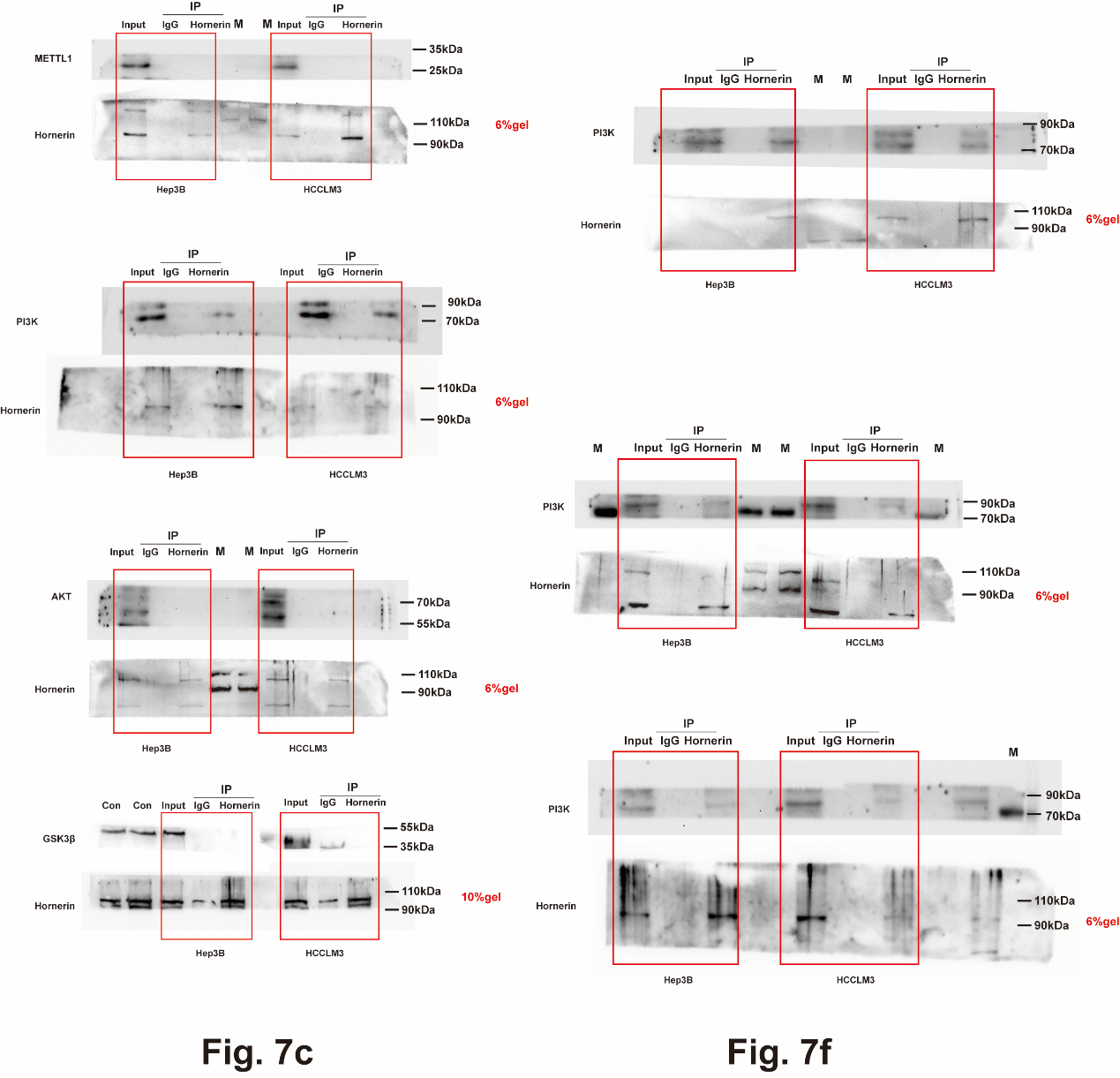

Supplement: Supplementary file 2 — wb original data [file 41419_2024_7248_MOESM2_ESM.docx]
